# *NF1* deficiency correlates with estrogen receptor signaling and diminished survival in breast cancer

**DOI:** 10.1038/s41523-018-0080-8

**Published:** 2018-08-30

**Authors:** Patrick S. Dischinger, Elizabeth A. Tovar, Curt J. Essenburg, Zachary B. Madaj, Eve E. Gardner, Megan E. Callaghan, Ashley N. Turner, Anil K. Challa, Tristan Kempston, Bryn Eagleson, Robert A. Kesterson, Roderick T. Bronson, Megan J. Bowman, Carrie R. Graveel, Matthew R. Steensma

**Affiliations:** 1 Center for Cancer and Cell Biology, Van Andel Research Institute, Grand Rapids, MI USA; 2 Bioinformatics & Biostatistics Core, Van Andel Research Institute, Grand Rapids, MI USA; 3 Department of Genetics, The University of Alabama at Birmingham, Birmingham, AL USA; 4 Vivarium and Transgenics Core, Van Andel Research Institute, Grand Rapids, MI USA; 5 Rodent Histopathology Core Dana Farber/Harvard Cancer Center, Harvard Medical School, Boston, MA USA; 6 Helen DeVos Children’s Hospital, Spectrum Health System, Grand Rapids, MI USA; 7 Michigan State University College of Human Medicine, Grand Rapids, MI USA

## Abstract

The key negative regulatory gene of the RAS pathway, *NF1*, is mutated or deleted in numerous cancer types and is associated with increased cancer risk and drug resistance. Even though women with neurofibromatosis (germline *NF1* mutations) have a substantially increased breast cancer risk at a young age and *NF1* is commonly mutated in sporadic breast cancers, we have a limited understanding of the role of *NF1* in breast cancer. We utilized CRISPR–Cas9 gene editing to create *Nf1* rat models to evaluate the effect of *Nf1* deficiency on tumorigenesis. The resulting *Nf1* indels induced highly penetrant, aggressive mammary adenocarcinomas that express estrogen receptor (ER) and progesterone receptor (PR). We identified distinct *Nf1* mRNA and protein isoforms that were altered during tumorigenesis. To evaluate *NF1* in human breast cancer, we analyzed genomic changes in a data set of 2000 clinically annotated breast cancers. We found *NF1* shallow deletions in 25% of sporadic breast cancers, which correlated with poor clinical outcome. To identify biological networks impacted by *NF1* deficiency, we constructed gene co-expression networks using weighted gene correlation network analysis (WGCNA) and identified a network connected to *ESR1* (estrogen receptor). Moreover, *NF1*-deficient cancers correlated with established RAS activation signatures. Estrogen-dependence was verified by estrogen-ablation in *Nf1* rats where rapid tumor regression was observed. Additionally, *Nf1* deficiency correlated with increased estrogen receptor phosphorylation in mammary adenocarcinomas. These results demonstrate a significant role for *NF1* in both *NF1*-related breast cancer and sporadic breast cancer, and highlight a potential functional link between neurofibromin and the estrogen receptor.

## Genetics: Mutant tumor suppressor linked to estrogen receptor signaling

Mutations in a tumor suppressor gene called *NF1* may be an important prognostic indicator for women with breast cancer and a therapeutic target for tumors resistant to hormone therapy. A team led by Carrie Graveel and Matthew Steensma from the Van Andel Research Institute in Grand Rapids, Michigan, USA, studied a large dataset of well-characterized breast cancer cases. They showed that 25% harbored mutations in *NF1*, a genetic alteration that correlated with diminished survival. Gene network analyses revealed links between *NF1* deficiency, *RAS* oncogene activity, and signaling through the estrogen receptor, including with genes known to mediate resistance to hormone therapy. The researchers also describe a newly created rat model of *NF1*-mutant breast cancer that they say could help further interrogate the importance of these genetic connections.

## Introduction

Deregulated RAS signaling promotes several “hallmarks of cancer”, such as sustained proliferation, invasion, metastasis, and angiogenesis.^1^ The importance of RAS deregulation in cancer is demonstrated by the fact that *KRAS* is the most commonly mutated oncogene and occurs at a high frequency in lung (17%), pancreatic (57%), and colon (33%) cancers.^2^ Even though KRAS is mutated in only 4% of sporadic breast cancers (*HRAS* = 1%; *NRAS* = 2%),^2^ the RAS/ERK pathway is hyperactivated in approximately 50% of breast cancers.^3–5^ This discrepancy suggests that there is another mechanism underlying RAS activation, besides mutation, in human breast cancers.

The key negative regulatory gene of the RAS pathway, *NF1*, is mutated or deleted in a wide range of cancers and is increasingly recognized as a significant cancer driver. The *NF1* gene encodes neurofibromin, a GTPase-activating protein that regulates RAS (including HRAS, NRAS, and KRAS). Loss of neurofibromin function results in hyperactivated RAS.^6^ Neurofibromin is a large 2818 amino acid protein in which exons 20–27 encode for the GAP-related domain (GRD).^7^ Neurofibromin functions as a monomer and stimulates intrinsic RAS GTPase activity through Spred1-mediated binding to the GRD in K-RAS, N-RAS, and H-RAS. Mutations or deletions within the GRD result in decreased neurofibromin functionality and deregulated RAS signaling driven through the RAS–RAF–MEK–ERK cascade.^6,8^ Neurofibromin contains other functional domains; however, the role of these domains and others in tumor suppression is undefined.^8^

Neurofibromatosis type 1 (NF) is caused by germline mutations in the *NF1* gene and is the most common single-gene disorder affecting 1 in 3000 live births.^9,10^ The majority of NF patients develop benign cutaneous neurofibromas and may also develop peripheral nerve tumors, neurocognitive disorders, and bone stigmata (tibial dysplasia, scoliosis, and osteoporosis). Importantly, NF patients have an increased risk of developing several adult cancers including breast, ovarian, liver, lung, bone, thyroid, and gastrointestinal.^11^ These diverse clinical manifestations reveal the impact of loss of NF1 function and dysregulated RAS in numerous tissue types.

In addition to germline mutations, *NF1* mutations and deletions commonly occur in sporadic cancers and are associated with increased cancer risk and drug resistance. *NF1* is the third most prevalent mutated or deleted gene in glioblastoma,^12^ fourth most mutated gene in ovarian cancer,^13^ and the second most common mutated tumor suppressor in lung adenocarcinoma.^14^ More recently, clinical evidence has mounted demonstrating that women with NF have a significantly increased breast cancer risk. A risk analysis of 3672 NF patients found that women with NF have an increased relative risk of developing breast cancer in their younger years compared to the general population (relative risk was 6.5 at 30–39 years, 4.4 at 40–49 years, and 2.6 at 50–59 years).^15^ Another key study of 1,404 NF patients identified an unequivocal increased risk for breast cancer with standardized incidence ratios of 11.1 (95% CI, 5.56–19.5) for breast cancer in women with NF age <40 years and the overall breast cancer mortality ratio was 5.20 (95% CI, 2.38–9.88).^16^ This study, in addition to several others, has established the increased breast cancer risk and associated poor outcome in patients with neurofibromatosis.^17^ Comprehensive genomic analyses of sporadic breast cancers revealed that *NF1* is commonly mutated and may be an important driver in sporadic breast cancer.^18,19^ A study using the *Mcm4*^*Chaos3*^ mouse model of chromosomal instability identified *Nf1* deletions in almost all of the *Mcm4*^*Chaos3*^ mammary adenocarcinomas.^20^ These findings suggest that *NF1* is a critical tumor suppressor and potential driver of breast tumorigenesis.

Much of our understanding of the mechanisms underlying the functional loss of *NF1* and tumorigenesis come from studies of genetically engineered mouse models.^21–24^ These models have been valuable in defining how *NF1* loss and deregulated RAS/MAPK signaling promote tumorigenesis. However, mouse models have general limitations, particularly with respect to reproducing human pharmacokinetics and recreating putative interactions between genetic/environmental factors and induced gene deficiency.^25^ It is well established that mice with germline heterozygous *Nf1* mutations do not spontaneously express key aspects of the human phenotype and require additional crosses into other germline mutants such as *Tp53*, or cell-specific conditional *Nf1* mutation, to elicit more representative phenotypes.^22,26–30^ Prior to the demonstration of CRISPR–Cas9 gene editing, manipulation of the rat genome was far more difficult than the mouse. The rat offers significant advantages over the mouse because of its larger size, more representative physiology to human disease, higher degree of cognition and memory, and ease of use in pharmaceutical studies.^31,32^ In our study we utilized CRISPR–Cas9 gene editing capabilities to create multiple congenic models of *Nf1* deletions in order to evaluate the effect of *Nf1* deficiency on tumorigenesis. The resulting *Nf1* indels affected transcription by generating both in-frame and out-of-frame sequence variation. Both mutation types were associated with highly penetrant, aggressive mammary adenocarcinomas in multiple rat founder lines. Phenotype penetrance and expression extended through second generation breeding including mammary adenocarcinomas in both *Nf1-*deficient males and females.

To evaluate the impact of *NF1* in sporadic breast cancer we analyzed genomic changes in a large breast cancer data set composed of >2000 clinically annotated breast cancers. We found that *NF1* shallow deletions are present in 25% of sporadic breast cancers and correlated with poor clinical outcome.^33^ To identify biological networks impacted by *NF1* deficiency, we constructed gene co-expression networks using weighted gene correlation network analysis (WGCNA) and identified co-expression networks. A module associated with *NF1* shallow deletion contained several genes that are considerably important in both ER + breast cancers and endocrine resistance, including *ESR1* and *FOXA1*. Unsupervised hierarchical clustering revealed that breast cancers with *NF1* shallow deletions form a distinct cluster that correlate with estrogen receptor (ER)-negative breast cancer and RAS activation. We also validated estrogen dependence of *Nf1*-deficient breast cancers by ablating mammary tumors in our *Nf1* rat model by ovariectomy. These results demonstrated the significant role *NF1* plays in both *NF1*-related breast cancer and sporadic breast cancer. Moreover, this novel *Nf1* rat model is invaluable for interrogating the role of *NF1*, estrogen-dependent breast cancer, and deregulated RAS signaling in sporadic and inherited breast cancer.

## Results

### Establishing *Nf1* rat models using CRISPR–Cas9 endonucleases

**Fig. 1:**
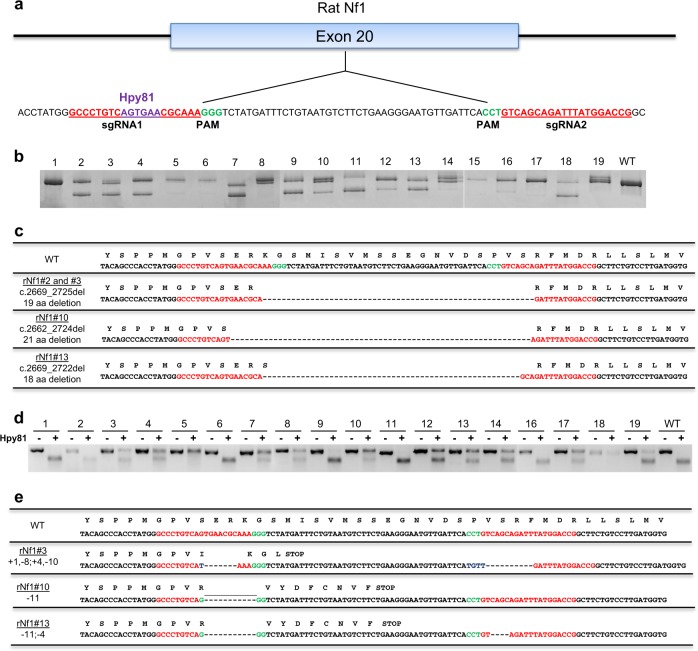
In-frame and premature indels are present in *Nf1* G0 rats. **a** CRISPR–Cas9 targeting of the *Nf1* GRD domain in exon 20 using two sgRNAs (red). PAM sequences (green) and the Hpy81 site (purple) used for HMA/restriction digest analysis are shown. **b** Heteroduplex PCR amplicons from 19 *Nf1* G0 animals. **c** DNA and peptide sequence alignment of the in-frame deletions within *Nf1* exon 20. **d** Heteroduplex PCR amplicons from 19 *Nf1* G0 animals were digested with Hpy81 to reveal the presence of smaller indels. No change between the −Hpy81 and +Hpy81 indicates the Hpy81 restriction digestion was lost during CRISPR recombination events. **e** DNA and peptide sequence alignment of the small indels at the 5′ and 3′ CRISPR target region

To investigate the effects of altered *Nf1* function in rat, we used two unique sgRNAs to target the GRD region in exon 20 and disrupt neurofibromin function (Fig. 1a). Two unique CRISPR/sgRNAs were synthesized and co-injected with Cas9 mRNA into one-cell-stage Sprague-Dawley rat embryos, which were then transferred to pseudopregnant females. From two rounds of injections, 19 pups were born. Genotyping was performed by amplifying a 452 bp fragment encompassing the sgRNA target sites from DNA isolated from tail biopsies, followed by a heteroduplex mobility assay (HMA).^34^ PCR amplicons with distinct HMA profiles were cloned into a plasmid vector and plasmids from multiple individual colonies were sequenced to identify the genetic lesions resulting from non-homologous end joining (NHEJ). Based on HMA profiles, indels were identified in 18 of 19 pups (Supplementary Tables 1 and 2). Multiple alleles were present in the majority of the G0 animals and were confirmed by Sanger sequencing, which revealed 34 mutant alleles, including 25 unique mutant alleles (Supplementary Table 2). Of the 34 mutations observed, 25 (73.5%) were frameshift mutations in both the 5′ and 3′ CRISPR target sites; 14 mutations (41.2%) were deletions ranging from 54–63 bp, spanning both the target sites. Interestingly, all the large deletions were in-frame with the translated protein coding sequence (Fig. 1b, c). HMA profiles of multiple G0 animals revealed the presence of additional, smaller indels at the 5′ and 3′ CRISPR target regions (Fig. 1b, i.e. lanes 3, 8, 10, etc) as have been observed in other studies.^34,35^ To verify the presence of the smaller indels and the larger in-frame deletions in each animal, we employed a three-step process of HMA, restriction digest, and Sanger sequencing (Fig. 1d, e, Supplementary Table 1, 2). The majority of smaller indels 10/19 (52.6%) were detected in the 5′ CRISPR target region of G0 animals. Within the 5′ CRISPR target region, there is a unique Hpy81 restriction site that is lost in each of the smaller indels. Consequently, PCR amplification and Hpy81 restriction digestion was used to confirm the presence of the smaller indels in each animal (Fig. 1d). Each of these small indels at the 5′ CRISPR target region resulted in premature stop codons in all cases (Fig. 1e, Supplementary Table 1). Indels were also observed in the 3′ CRISPR target region in 5/19 (26.3%) animals; however, these were not predicted to have an effect on protein translation due to the presence of premature stop codons at the 5′ CRISPR target region.

To investigate the effects of both the *Nf1* in-frame (referred to as IF) and premature stop (referred to as PS) indels, we bred four founder rats (2 male and 2 female) that carried a mix of indels consisting of in-frame deletions and premature stops with wild-type Sprague–Dawley rats (Fig. 1 and Supplementary Table 1). From two male founders, we generated two unique lines: *Nf1*^*IF-57/+*^ from male founder #2 and *Nf1*^*IF-57/PS-8*^ derived from male #3. From two female founders, the lines *Nf1*^*IF-63/PS-11*^ (founder #10) and *Nf1*^*IF-54/PS-11*^ (founder #13) were generated. Segregation of the IF and PS alleles occurred in the G1 generations for lines *Nf1*^*IF-57/PS-8*^ and *Nf1*^*IF-54/PS-11*^, resulting in *Nf1*^*PS-8/+*^, *Nf1*^*PS-11/+*^, *Nf1*^*IF-57/+*^ lines (male #3). These *Nf1* lines were bred out for the following studies.

Upon performing PCR-HMA and sequence analysis, we observed the presence of more than two *Nf1* alleles in individual animals. In *Nf1*^*IF-63/PS-11*^ we identified 3 *Nf1* alleles: a WT allele, an allele with an 11 bp deletion (premature stop), and an allele with a −63 bp deletion (Supplementary Fig. 1, Supplementary Table 1). The presence of more than 2 alleles was confirmed independently by HMA and sequencing analysis using unique primer sets in two separate labs. Furthermore, these 3 alleles were transmitted through the germline in G1 and G2 generations and did not segregate in either generation (Supplementary Fig. 1). Even though this finding was unanticipated, this is not unique in that the rat genome is known to have additional allelic copies of other genes.^36,37^

### *Nf1* female and male rats develop aggressive mammary adenocarcinomas

*Nf1* rats (including in-frame and premature stop indels) were aged to determine the effects of *Nf1* alterations on tumor development. In *Nf1* female rats, we observed aggressive, multifocal tumorigenesis in the mammary glands at a young age (6–8 weeks). Mammary tumors were observed in 100% (11/11) of G0 females at the average age of 51 days (Supplementary Table 1). Tumor development progressed rapidly in all but one female (*rNf1*#19), whereas the majority of G0 females had to be euthanized before reaching a mature breeding age. We were able to breed two females before their tumor burden required euthanasia (*rNf1*#10 and #13) and support the pups through fostering. Multiple mammary adenocarcinomas (2–8) were observed in each animal and were detected in mammary glands 1–6. The detailed tumor pathology for each animal is described in Supplementary Table 3. Notably, two male founders (*rNf1*#2 and #3) developed mammary tumors at 14–16 months of age. Male #2 developed four unique mammary adenocarcinomas, whereas male #3 developed one mammary adenocarcinoma but had to be killed due to a larger fibrous histiocytic sarcoma. This aggressive mammary phenotype was highly penetrant and multifocal mammary tumors were observed in the subsequent G1 and G2 generations from each of the *Nf1* lines, including lines derived from male founders (Supplementary Table 3).

**Fig. 2:**
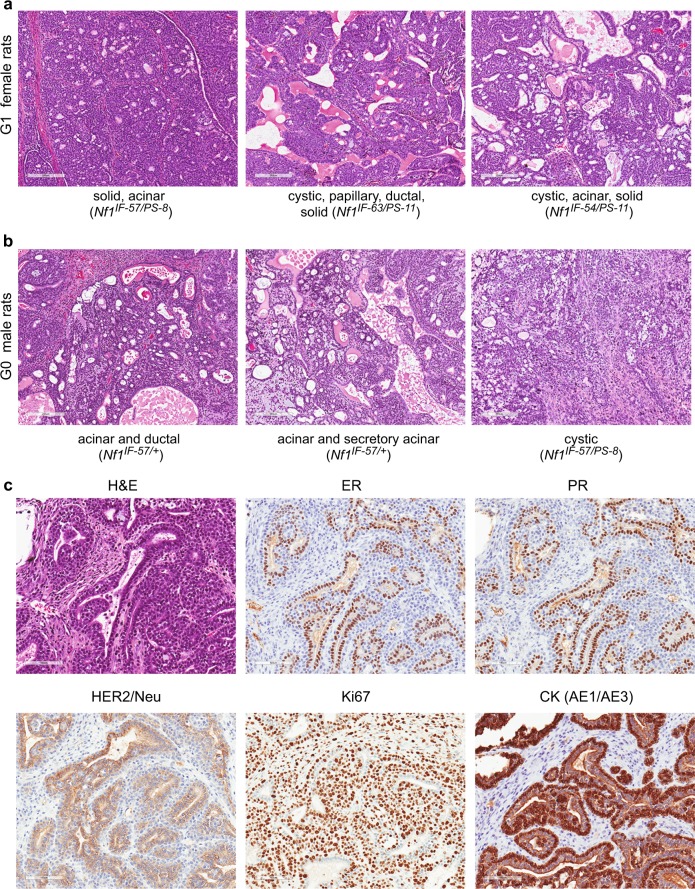
*Nf1* deficiency induces ER^+^/PR^+^ mammary tumors. We observed mammary tumors with diverse mammary histology in G0 - G2 animals. **a** *Nf1* females and **b** *Nf1* males developed mammary adenocarcinomas with mixed histology including acinar, cystic, papillary, ductal, and solid features. All images were taken at 100X magnification. **c** Immunostaining of *Nf1* mammary tumors (*rNf1* #6) for estrogen receptor, progesterone receptor, HER2, Ki67, and pan-cytokeratin (AE1/AE3). All immunostaining images were taken at 200X magnification

Histopathologic analysis of the mammary tumors revealed that both *Nf1* in-frame deletions and premature stop indels induced a wide variety of histopathologic mammary tumor types. Mammary tumors with acinar, solid, ductular, and cystic histology were observed in tumors in *Nf1* female rats from each line (Fig. 2a). Moreover, *Nf1* animals often developed mammary adenocarcinomas with mixed histology such as acinar and solid (Fig. 2a, left panel); cystic, papillary, ductal, and solid (Fig. 2a, middle panel); and cystic, acinar, and solid (Fig. 2a, right panel). This diverse mammary histology was observed in G0, G1, and G2 animals. In addition, we observed mixed histopathology in the mammary adenocarcinomas from male *Nf1* rats (Fig. 2b). The level of tumor burden in mammary pads of several animals was substantial, as can be observed in *rNf1* #413 (Supplementary Table 3). As shown in Supplementary Fig. 2, one mammary gland from *rNf1* #413 contained 6 separate mammary tumors (2 additional larger tumors were separated for histopathology). Immunostaining of several tumors revealed that the *Nf1* mammary tumors were positive for estrogen receptor, progesterone receptor, and HER2/Neu receptor and highly proliferative based on Ki67 staining (Fig. 2c). We compared proliferation in the *Nf1*^*IF*^ and *Nf1*^*PS*^ tumors with Ki67 staining (Supplementary Fig. 3). Even though we observed diversity in proliferation among tumors, quantitative image analysis found no statistically significant difference. The results demonstrate the impact of *Nf1* loss of function on mammary tumor initiation in both male and female hormonal environments.

### *Nf1* premature stop indels result in more aggressive initiation and tumor progression

**Fig. 3:**
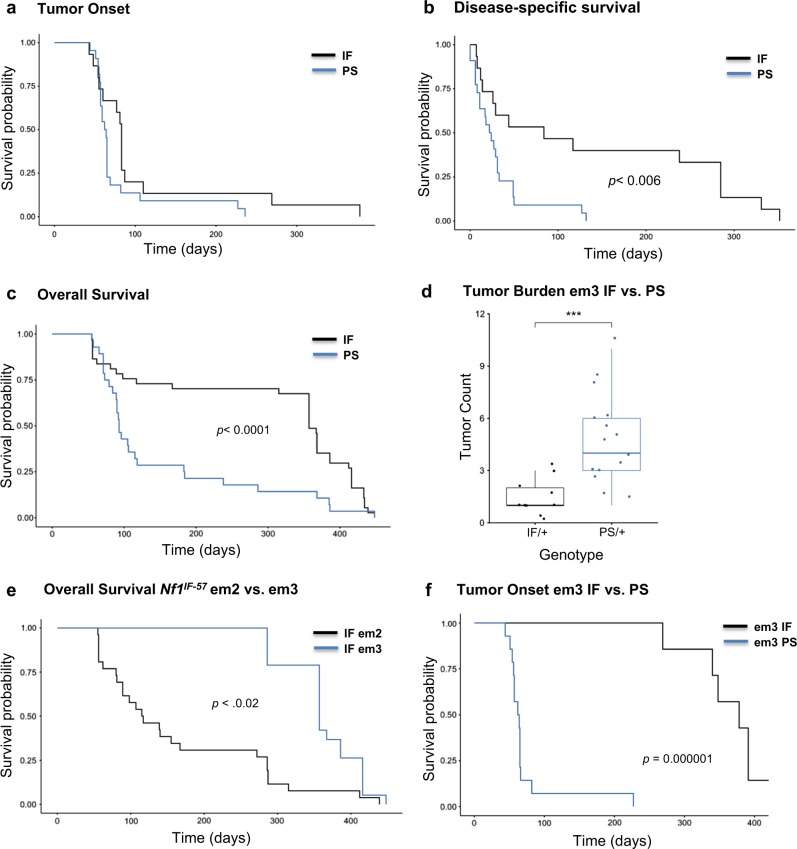
Survival analysis of *Nf1*^*IF*^ vs. *Nf1*^*PS*^ reveals distinct effects of *Nf1* deficiency on tumor onset and survival. Survival analysis was performed on *Nf1*^*IF*^ (n = 35) vs. *Nf1*^*PS*^ (*n* = 24) in the *Nf1*^*IF-57/+*^*, Nf1*^*IF-57/PS-8*^*, Nf1*^*IF-54/PS-11*^ lines. Kaplan–Meier plots of **a**) tumor onset, **b** disease-specific survival, and **c** overall survival are shown. **d** Tumor burden of em3-*Nf1*^*IF-57/+*^ compared to em3-*Nf1*^*PS-8*^ rats. **e** Overall survival analysis of em2 and em3 lines harboring the *Nf1*^*IF-57/+*^ indel showed a discrepancy in survival. **f** Tumor onset of em3-*Nf1*^*IF-57/+*^ compared to em3-*Nf1*^*PS-8*^ rats. All animals in this study died of mammary tumor progression

To evaluate how in-frame versus premature *Nf1* indels affect disease burden and survival, we examined the overall survival of G1 heterozygous females. We performed a Kaplan–Meier analysis on overall survival of *Nf1*^*IF*^ (*n* = 35) compared to *Nf1*^*PS*^ (*n* = 24) in three rat lines (*Nf1*^*IF-57/+*^*, Nf1*^*IF-57/PS-8*^*, Nf1*^*IF-54/PS-11*^). Survival analysis of tumor onset demonstrated that there was no statistical difference between the time of onset in premature stop compared to in-frame indels (Fig. 3a). Conversely, analysis of disease-specific survival (*p* < 0.006) and overall survival (*p* < 0.0001) revealed that *Nf1*^*PS*^ animals died due to tumor burden significantly faster than *Nf1*^*IF*^ animals (Fig. 3b, c). These data were influenced by a significant increase in tumors observed in *Nf1*^*PS*^ animals. As shown in Fig. 3d, *Nf1*^*PS*^ animals had an average of 3X more tumors than *Nf1*^*IF*^ animals (*p* = 0.00006, 95% CI [1.82, 5.41]). Notably, all animals in this study died of mammary tumor progression. We compared the overall survival of two *Nf1*^*IF-57*^ lines that were derived from separate founders (em2 and em3). Interestingly, we observed that the em2-*Nf1*^*IF-57*^ rats had a significantly shorter survival than em3-*Nf1*^*IF-57*^ rats, even though these *Nf1* lines had identical in-frame deletions (*p* < 0.02; Fig. 3e). The potential reason for this discrepancy in survival is described below.

### Distinct neurofibromin isoform is expressed in mammary tissue

**Fig. 4:**
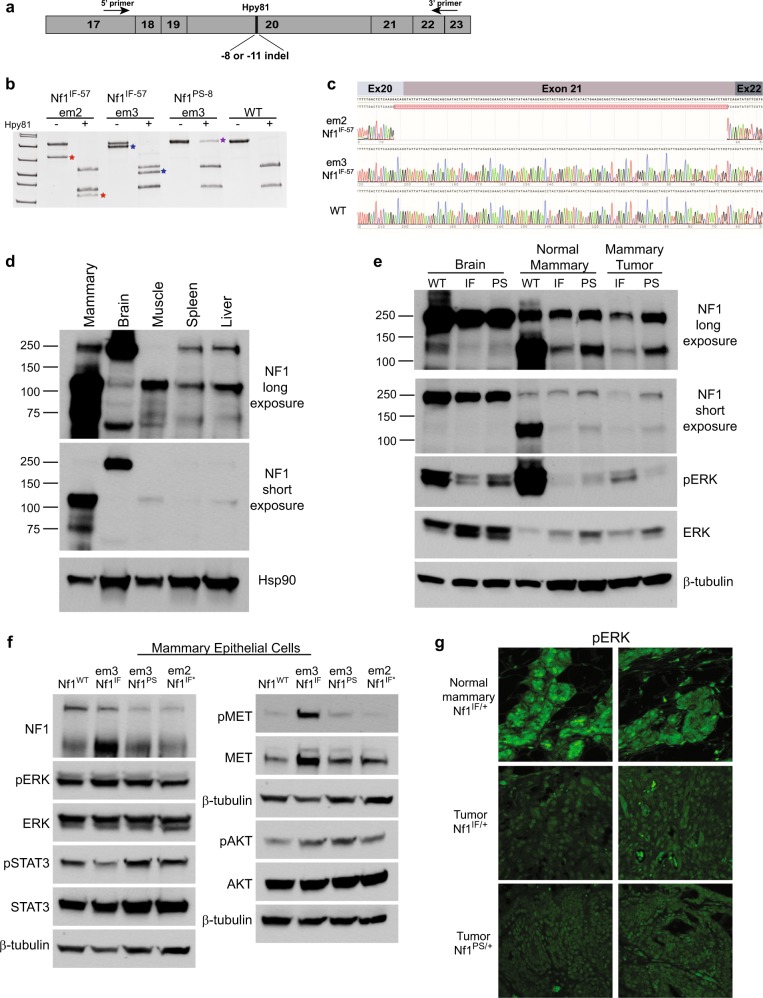
Analysis of *Nf1*^*IF*^ and *Nf1*^*PS*^ mRNA and protein isoforms. **a** Schematic of RT-PCR analysis of *Nf1* exons 17-23. **b** RT-PCR products were digested with Hpy81 and separated by PAGE to differentiate *Nf1*^*IF*^ and *Nf1*^*PS*^ mRNA. Unique bands were observed in the em2-*Nf1*^*IF*-57^ and em3-*Nf1*^*IF*-57^ REF cDNA (noted by red and blue stars). The PS mRNA that is not digested due to the loss of the Hpy81 site is noted (purple star). **c** Sequence analysis of em2-*Nf1*^*IF*-57^ and em3-*Nf1*^*IF*-57^ REF cDNA. **d**, **e** Immunoblot analysis showing **d** tissue-specific expression of rat neurofibromin isoforms and **e** differential expression of the neurofibromin 250 and 125 kD bands in matched brain, normal mammary tissue, and mammary tumor at both a long (top) and short (bottom) exposure. **f** Western blot analysis of neurofibromin, ERK, STAT3, MET, and AKT phosphorylation and expression in MECs derived from normal *Nf1* rat mammary pads. **g** Representative immunofluorescence of pERK1/2 in normal mammary tissue and mammary adenocarcinomas. Images are shown at ×600

To understand how *Nf1* deficiency via mutation of the GRD domain affects neurofibromin expression and activity, we first examined the *Nf1* isoforms that are expressed in distinct *Nf1*^*IF*^ and *Nf1*^*PS*^ lines. mRNA was isolated from day 13.5 rat embryos fibroblasts (REFs) and RT-PCR was performed using primers in exon 17 and exons 22–23 (Fig. 4a). To differentiate RT-PCR products derived from the WT, IF, and PS alleles, we performed restriction digests with the Hpy81 endonuclease (Fig. 4b). As demonstrated in Fig. 1d, e, alleles harboring premature stop indels have lost the Hpy81 restriction site at the 5′ CRISPR site. RT-PCR of WT REFs resulted in a strong band at 826 bp that created two bands at 390 bp and 368 bp with Hpy81 digestion. RT-PCR and Hpy81 digestion of the *Nf1*^*PS-8*^ fibroblasts resulted in *Nf1*^*WT*^ bands and an undigested band at 745 bp. Expression of the WT allele was observed in each of the *Nf1* in-frame and premature stop REF lines. We then compared the em2- *Nf1*^*IF-5*^ and em3-*Nf1*^*IF-57*^ lines that had distinct survival curves (Fig. 3e). Even though genotyping confirmed an identical 57 bp deletion in exon 20 in both lines, we observed distinct *Nf1* mRNA isoform expression patterns (Fig. 4b, notated with *). To determine the difference in the *Nf1*^*IF-57*^ mRNA isoforms, we cloned and sequenced each of the products. Overall, we sequenced seventeen clones where the majority contained the predicted *Nf1*^*IF-57*^ and *Nf1*^*WT*^ isoforms, but we identified 3 unique clones harboring exon 21 deletions or extended exon 20 deletions resulting in *Nf1* premature stops. For example, one mRNA isoform contained the 57 bp in-frame deletion in exon 20 and an additional 140 bp deletion in exon 21 (Fig. 4c). This exon 21 deletion mRNA was exclusive to the em2-*Nf1*^*IF-57*^ line and resulted in a premature stop that would impact the GRD domain and other downstream functional domains. Because our initial analysis of tumor onset (Fig. 3a) included the em2-*Nf1*^*IF-57*^ animals as “IF”, we repeated our statistical analysis of tumor onset in em3-*Nf1*^*IF-57*^ versus em3-*Nf1*^*PS-8*^ animals. As shown in Fig. 3f, the tumor onset was significantly earlier in *Nf1*^*PS*^ rats compared *Nf1*^*IF*^ animals. These results substantiate the requirement of a functional GRD domain within NF1 to suppress tumor progression.

Several unique isoforms have been characterized in the *Nf1* gene with the main isoform identified at 250 kD; however, the majority of these studies have been performed in brain, neuronal, and muscle tissues.^38–40^ To our knowledge, neurofibromin expression has not been previously evaluated in the mammary glands of humans or rodents. To understand the effect of *Nf1* deficiency in the mammary gland, we first performed Western blot analysis of neurofibromin in the normal mammary, brain, muscle, spleen, and liver of adult rats and observed distinct isoform expression patterns among the tissue types (Fig. 4d). Even though the established 250 kD neurofibromin isoform is highly expressed in brain tissue, its expression was significantly less in other tissues, including the mammary gland. In the mammary gland, the predominant neurofibromin isoform was observed at 125 kD. This 125 kD isoform was also observed in the muscle, spleen, and liver, yet minimally expressed in the brain. In Fig. 4e, we compared neurofibromin expression in the brain, normal mammary glands, and mammary adenocarcinomas from *Nf1*^*WT*^, *Nf1*^*IF*^, and *Nf1*^*PS*^ rats. In the brain, the 250 kD isoform was highly and equally expressed in the *Nf1*^*WT*^, *Nf1*^*IF*^, and *Nf1*^*PS*^ animals. In the normal mammary gland, there was no difference in expression of the 250 kD isoform between the *Nf1*^*WT*^, *Nf1*^*IF*^, and *Nf1*^*PS*^ animals, however there was a substantial decrease in expression of the 125 kD isoform in both the *Nf1*^*IF*^ and *Nf1*^*P*S^ mammary glands compared to WT mammary glands. In mammary adenocarcinomas, 125 kD isoform was further decreased in both the *Nf1*^*IF*^ and *Nf1*^*PS*^ tumors and there appeared to be a slight loss of the 250 kD isoform in the *Nf1*^*IF*^ mammary tumor. Overall these results demonstrate the distinct neurofibromin isoform tissue preferences that are present and the inverse relationship of the 250 kD vs. 125 kD isoforms in the brain and mammary tissues.

To determine the effect of *Nf1* deficiency on RAS signaling, we examined ERK staining in normal mammary tissue, mammary epithelial cells, and mammary tumors. In normal mammary glands we observed a very strong pERK signal that was unexpectedly lower in the *Nf1*^*IF*^ and *Nf1*^*PS*^ normal mammary and tumor tissue (Fig. 4e). In normal brain tissue where the 250 kD neurofibromin isoform is predominantly expressed, total ERK was increased in the *Nf1*^*IF*^ and *Nf1*^*PS*^ brains compared to *Nf1*^*WT*^, yet pERK was strongest in the *Nf1*^*WT*^ tissue (Fig. 4e). Given that the mammary pad is composed of primarily stromal cells (i.e., adipocytes, fibroblasts, immune cells), we wanted to evaluate the effect of *Nf1* deficiency specifically in the mammary epithelium. We optimized a procedure to isolate mammary epithelial cells (MECs) from normal rat mammary fat pads and examined *Nf1* expression and signaling in *Nf1*^*WT*^, *Nf1*^*IF*^, and *Nf1*^*PS*^ MECs (Fig. 4f). In the MECs, we observed distinct differences in neurofibromin expression and signaling compared to the whole mammary tissue. In the *Nf1*^*WT*^ MECs, both the 250 kD and 125 kD isoforms were expressed and in the em3-*Nf1*^*IF*^ MECs, expression of the 125 kD neurofibromin isoform was moderately increased. In contrast, in *Nf1*^*PS*^ and em2-*Nf1*^IF*^ (which express the additional exon 21 mRNA deletion and mimic the *Nf1*^*PS*^) MECs, neurofibromin 250 kD expression was decreased. This decrease in neurofibromin expression resulted in no change in total ERK or pERK compared to *Nf1*^*WT*^ (Fig. 4f). Next, we assessed pERK subcellular localization using immunofluorescence and observed cytoplasmic pERK in the normal mammary pad (Fig. 4g; Supplementary Fig. 4). In contrast, we observed predominantly nuclear pERK in tumors from *Nf1*^*IF*^ and *Nf1*^*PS*^ animals (Fig. 4g; Supplementary Fig. 4a). When we compared pERK staining in the tumor periphery compared to the interior, we observed a moderate increase in ERK activity at the tumor periphery (Supplementary Fig. 4b). We also examined AKT, MET, and STAT3 which we and others have shown to be critical signaling pathways involved in the progression of NF1-related sarcomas.^41,42^ For the MET receptor we observed an increase in both activated (phosphorylated MET) and total MET in only the *Nf1*^*IF*^ MECs (Fig. 4f), yet immunostaining revealed an increase in pMET in both *Nf1*^*IF*^ and *Nf1*^*PS*^ tumors compared to normal mammary tissue (Supplementary Fig. 5a). There was no change in AKT expression or phosphorylation in the MECs or tumors, yet we observed an increase in both pSTAT3 expression and nuclear localization in both the *Nf1*^*IF*^ and *Nf1*^*PS*^ MECs and tumors (Fig. 4f; Supplementary Fig. 5b). These results indicate that several of the RAS linked signaling pathways altered in other NF-related cancers are altered in *Nf1*-mediated mammary tumor progression.

### *NF1* is commonly deleted in human sporadic breast cancer and correlates with ER networks

**Fig. 5:**
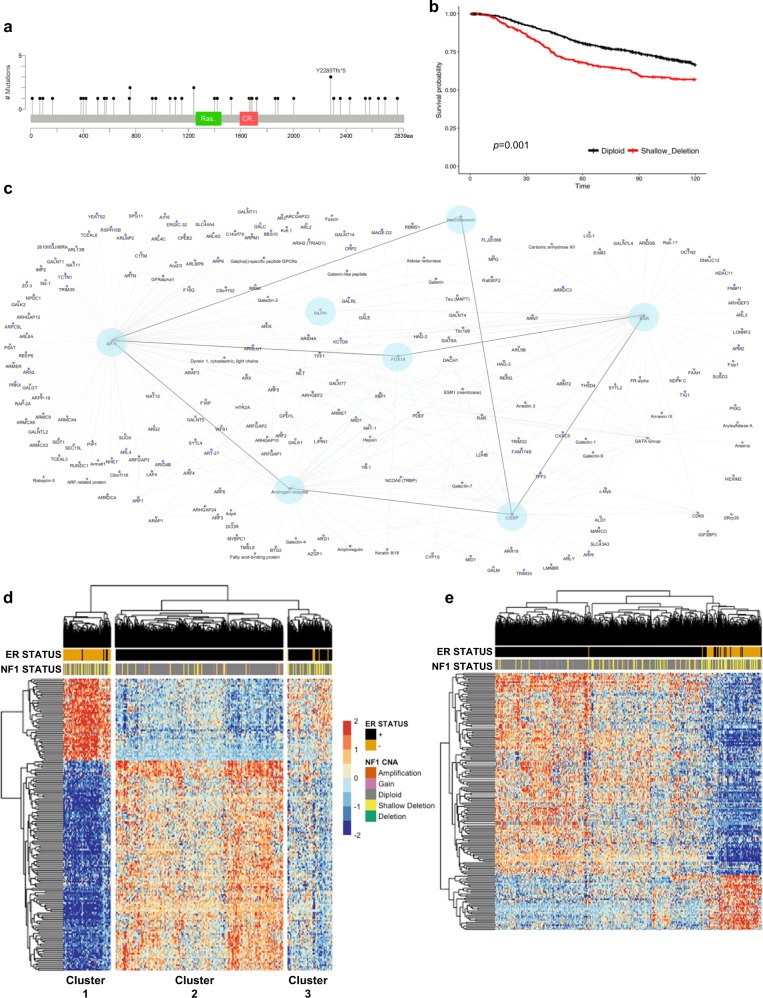
*NF1* shallow deletions are frequently present in sporadic breast cancers and associate with *ESR1* and *FOX1A*. **a** Truncating mutations were detected throughout *NF1* in METABRIC patients (cBioportal). **b** Survival analysis of breast cancer patient survival comparing *NF1* diploid and shallow deletion copy number status. **c** Network visualization of WGCNA module genes associated with *NF1* shallow deletion copy number status. **d** Heatmap of METABRIC gene expression data of WGCNA module genes correlated with *NF1* copy number and ER status. **e** Heatmap of METABRIC gene expression data of WGCNA module genes associated with RAS activation signatures

Even though it is known that neurofibromatosis patients have a significantly increased breast cancer risk and that *NF1* is mutated in sporadic breast cancers, the impact of *NF1* deficiency and RAS deregulation in sporadic breast cancer is often overlooked. To interrogate the impact of *NF1* loss in breast cancer, we performed an analysis of the METABRIC breast cancer data set which contained 2,051 patients with clinical annotation including CNV and SNP genotypes.^33^ In this cohort, there were 43 patients (2.1%) with truncating mutations, including frameshift deletions or insertions, nonsense mutations, and splice site alterations (Fig. 5a). Notably, these mutations occurred throughout the *NF1* gene similar to the mutation diversity observed in NF patients. After removing patients with *NF1* SNP mutations, we analyzed *NF1* copy number alterations (CNAs) and identified CNAs in 32.9% of patients, with 24.5% having *NF1* shallow deletions (defined as potential heterozygous deletions). To determine the effect of *NF1* shallow deletions on survival, we conducted a survival analysis using the Cox proportional hazards model adjusting for ER status and age at diagnosis. We found that patients with an *NF1* shallow deletion had significantly higher tumor grade, stage, and size (Supplementary Table 4). They were also more likely to have basal-like breast cancer (*p* < 0.0001) and less likely to have luminal A breast cancers (*p* < 0.0001). Moreover, patients with an *NF1* shallow deletion had a hazard ratio of 1.4 (*p* = 0.001) relative to diploid *NF1* status for 10 year survival (Fig. 5b, Supplemental Table 4).

To identify gene expression networks that correlate with *NF1* copy number status, we utilized weighted gene co-expression network analysis (WGCNA).^43^ From the METABRIC data set, expression data were analyzed for patients with available copy number and gene expression data (*n* = 1427). Since *NF1* is in close proximity to *HER2* on chromosome 17 and is often co-amplified, we removed HER2-amplified patients from this analysis. From the 18,049 genes in the METABRIC data set, 2,218 were placed in 12 co-expression modules. Modules contained between 30 and 500 genes, with an average module size of 200 genes. Interestingly, one module (179 genes) associated with *NF1* shallow deletion copy number status contained several genes that are considerably important in both ER+ breast cancers and endocrine resistance, including *ESR1* (Fig. 5c). Hub genes for this module, or genes with high connectivity, included *FOXA1* and *MLPH*. *FOXA1*, a Forkhead family transcription factor, regulates ER binding and transcriptional activity.^44^ *FOXA1* expression correlates with luminal subtype A breast cancer and is a significant predictor of cancer-specific survival in ER+ tumors.^45^ *MLPH* expression is also associated with longer survival in breast cancer.^46^ Further network visualization of genes within this ER-associated co-expression network revealed additional connections with *AP1*, *AR* (androgen receptor) and *C/EBPα* (CCAAT/enhancer binding protein). AP1 is also known as the transcription factor JUN. RAS/ERK signaling is known to regulate JUN at the transcriptional and post-translational (phosphorylation) level and can modify lysine acetylation in JUN DNA binding regions.^47^ Recent studies have demonstrated that *AR* is expressed in 77% of breast cancers (88% ER+, 59% HER2+, 32% TNBC)^48^ and is involved in endocrine resistance in ER+ breast cancers.^49,50^ Although there are studies demonstrating interaction of *C/EBPα* at ER transcriptional binding sites, the consequence of *C/EBPα* activity in breast cancer is unclear.

Unsupervised clustering of genes from this WGCNA module with METABRIC clinical data revealed a strong association of *NF1* shallow deletions in both ER+ and ER- tumor subsets. This analysis distinguished three modules associated with *NF1* copy number status, with one module comprised of 181 genes associated specifically with *NF1* shallow deletions (Fig. 5d). The heatmap in Fig. 5d revealed three clusters associated with NF1 and ER status: Cluster 1 (primarily ER-), Cluster 2 (ER+), and Cluster 3 (primarily ER+). The odds of Cluster 1 patients having an *NF1* shallow deletion (versus another mutation) is 2.01 times higher than Cluster 3 and 5.55 times higher than Cluster 2 (*p* = 0.0002 and *p* < 0.0001; 95% CI [1.39, 2.89] and 4.08, 7.63], respectively). Whereas, the odds of Cluster 3 having a shallow deletion is 2.76 times higher than Cluster 2 (*p* < 0.0001; 95% CI [1.99, 3.84]). To understand if *NF1* shallow deletions correlate with deregulated RAS signaling, we employed expression signatures developed from KRAS-mutant cancers and performed a secondary WGCNA analysis and unsupervised clustering with 788 genes related to ER, RAS and KRAS signaling pathways (Fig. 5e). These results validate the presence of RAS activation in *NF1*-related breast cancers. Moreover, our WGCNA analyses revealed novel connections between *NF1* deficiency, RAS signaling, and ER signaling.

### *Nf1*-deficient tumors are estrogen-dependent

**Fig. 6:**
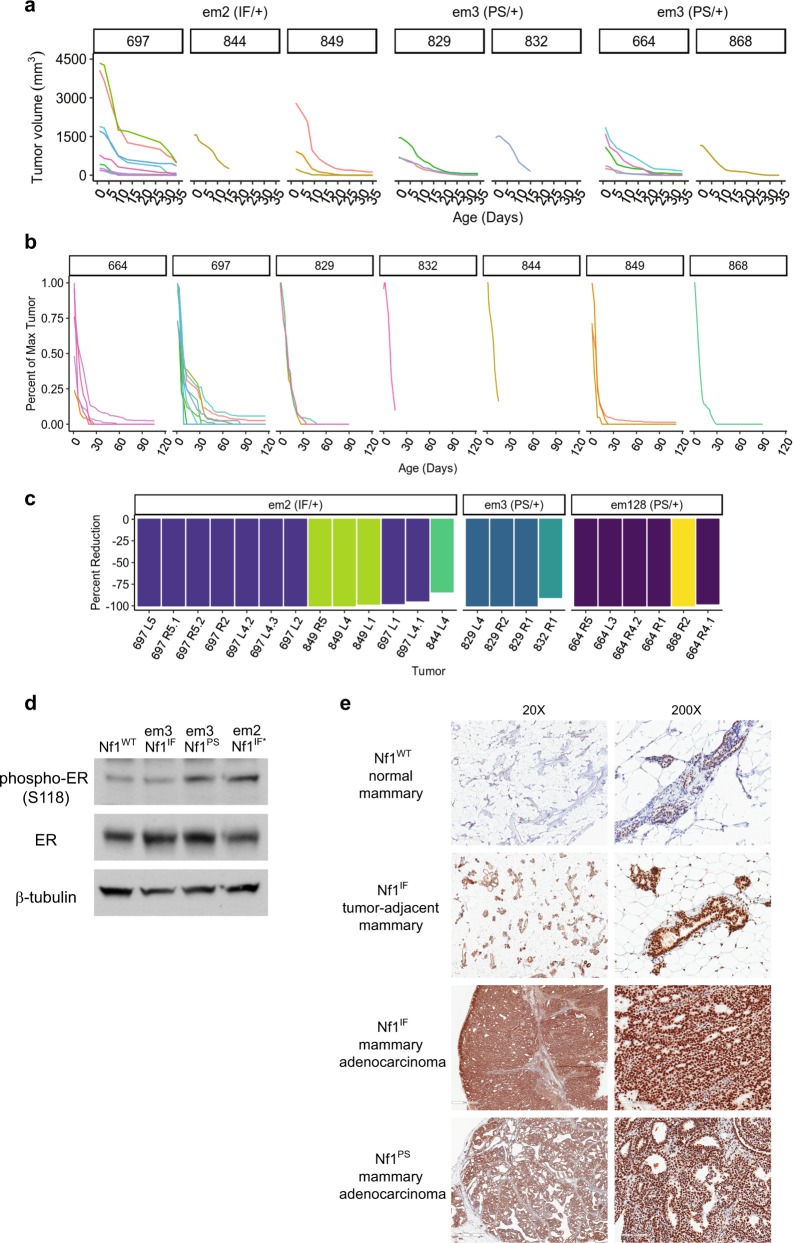
Estrogen depletion via ovariectomy results in rapid tumor regression in *Nf1*-deficient tumors. **a** Tumor volume was plotted after ovariectomy (Day 0) for individual *Nf1*^*IF*^ and *Nf1*^*PS*^ rats. **b** Percent tumor change over time of multiple tumors in individual *Nf1*^IF^ and *Nf1*^PS^ rats after ovariectomy (Day 0). **c** The percent reduction in tumor size for all tumors from 3 *Nf1* rat lines is shown. Distinct colors are used to represent individual rats and the mammary pad location of each tumor is notated. Note that several rats had multiple measurable tumors within an individual mammary pad. **d** Immunoblot analysis of phospho-ER (S118) and ER expression in MECs derived from normal *Nf1* rat mammary pads. **e** Immunostaining of phospho-ER (S118) in normal mammary tissue and mammary adenocarcinomas from *Nf1*^*WT*^, *Nf1*^*IF*^ and *Nf1*^*PS*^ rats

To examine whether our *Nf1*-deficient rat breast cancer model was estrogen dependent, we performed ovariectomies on rats containing at least one tumor greater than 1500 mm^3^. Ovariectomies were performed on 7 rats with multiple tumors from three distinct *Nf1* lines (Fig. 6a–c). Upon ovary removal, tumor size diminished at a rapid pace (mean reduction per day = 4.7% per day (*p* < 0.0001; 95% CI [3.8, 5.5]). Tumor reduction was observed in all of the tumors regardless of *Nf1* status (in-frame vs. premature stop indels) and the mean total percent reduction in tumor volume was 97.8% (*p* < 0.0001; 95% CI [94.1, 99.8]) (Fig. 6c). ER is tightly regulated through numerous post-translational modifications including phosphorylation, acetylation, methylation, sumoylation, and ubiquitination. Previously it has been demonstrated that ERK can phosphorylate ER*α* at S104/106 and S118, where S118 phosphorylation facilitates ER*α* interactions with coactivators.^51,52^ We examined ER*α* expression and S118 phosphorylation in MECs and observed no change in total ER, but p-ER*α* was increased in both *Nf1*^*PS*^ and *Nf1*^IF*^ MECs (Fig. 6d). This pattern matched the level of decrease in neurofibromin expression observed in Fig. 4f. When we examined p-ER*α* (S118) in normal mammary tissue from an *Nf1*^*WT*^ rat, we observed weak to moderate staining in the mammary epithelial cells (Fig. 6e). In contrast, mammary adenocarcinomas from both *Nf1*^*IF*^ and *Nf1*^*PS*^ animals had extremely intense ER*α* pS118 staining. Strong p-ER*α* was also observed in *Nf1*^*IF*^ mammary tissue adjacent to a tumor in comparison to the *Nf1*^*WT*^ mammary tissue. These results confirm estrogen-dependency in the rat *Nf1-*deficient breast cancer model and support the biological significance of the NF1-ER networks that were identified by WGCNA.

## Discussion

The RAS signaling pathway is intricately involved in the initiation and progression of numerous cancer types; however, the mechanisms by which RAS is deregulated in breast cancer are unclear. Here we present data that illuminates the role that *NF1* plays in breast cancer and a novel model for interrogating the interaction between NF1 and ER in both sporadic and NF-related breast cancer. Even though NF patients are predisposed to variety of cancers, breast cancer risk was not confirmed until recently.^15–17^ *NF1* was first implicated in sporadic breast cancer by the initial comprehensive genome breast cancer studies where *NF1* mutations were identified in 2–4% of breast cancers.^18,19^ Our analysis of the extensive METABRIC data set verified the *NF1* mutation frequency, but also discovered *NF1* shallow deletions in 25% of sporadic breast cancers. The analysis also revealed *NF1* shallow deletions correlate with higher tumor grade, tumor size, and the aggressive, basal subtype. Most importantly, *NF1* shallow deletions correlate with poor outcome within the first 10 years. Thus, our work strengthens the conclusion drawn from the TCGA breast cancer data set and the role of *NF1* as a bona-fide driver.^20^ In addition to *NF1*, the RasGAP gene *RASAL2* is altered in sporadic breast cancer. McLaughlin et al. demonstrated that *RASAL2* expression is substantially decreased in luminal B breast cancers and low *RASAL2* expression correlates with metastasis and poor survival.^53^ Our analysis extends these findings towards the basal subtype which exhibits a disproportionately increased number of *NF1* shallow deletions. Taken together, these studies indicate that loss of RAS suppression may be more important in breast cancer than RAS activation through mutation or amplification.

Our WGCNA analysis revealed an unknown network between *NF1*, *ESR1*, and RAS signaling in breast cancer. In our *Nf1* tumor model, we observed strong ER (S118) phosphorylation indicating a role for indirect effects of *Nf1* deficiency through ERK phosphorylation of the S118 motif. However, recent findings demonstrate direct NF1-ER binding that is mediated by the ER ligand binding domain and NF1 leucine-rich regions.^54^ These exciting results indicate that NF1 acts like a classic ER corepressor and that corepressor activity requires NF1 GAP activity and the Leu-rich motif. Our results complement these findings and strongly suggest that *NF1* deficiency directly and indirectly impacts ER signaling. Interrogating the mechanisms by which NF1 interacts with ER will require a careful analysis of both transcriptional and signaling interactions in various hormonal environments. Neurofibromin is a large protein and there is a limited knowledge of neurofibromin protein-protein interactions besides the RAS interactions at the GRD domain. Even though neurofibromin may directly interact with genes involved identified in the NF1-ER network, indirect NF1-RAS-ER signaling interactions are likely since RAS signaling regulates JUN (AP1) at the transcriptional and post-translational level.^47^ It is also possible that direct neurofibromin-ER interactions are isoform dependent. Using our *Nf1* rat model, human breast cancer lines, and patient-derived xenograft models, we will be able to interrogate how the presence and absence of estrogen impacts direct and indirect NF1-ER signaling.

Together, our CRISPR rat *Nf1* model and our WGCNA analysis of human breast cancer confirms that *NF1* is intricately connected to ER networks, including several genes that have been directly linked to endocrine resistance (i.e., *FOXA1* and *AR*). It is interesting to note that the rate of tumor regression in the *Nf1* tumors after estrogen ablation was remarkably rapid and included all *Nf1* tumors regardless of mutation (PS or IF). These data suggest that our *Nf1*-deficient tumors are universally estrogen-dependent. The WGCNA data indicate more distinct roles for *NF1* in both estrogen positive and negative environments. One potential explanation for these divergent results is that *Nf1*-deficient tumors are treatment naïve, occurring in younger animals, whereas adult tumors in the METABRIC data set are diverse with respect to treatment and host factors. A recent study showed that *NF1* mutations contribute to the genomic evolution towards metastasis and are prevalent in recurrent, ER + breast cancers.^55^ Considering our demonstration that (1) *NF1* shallow deletions correlate with poor outcome, (2) network connectivity exists between NF1-ER-FOXA1-AR, and (3) broad response of *Nf1* tumors to ovariectomy, it is likely that *NF1* is a key factor in endocrine resistance.

The *Nf1* rat model also revealed some unexpected alternative mRNA and protein isoforms that underscore our limited understanding of *NF1* expression. For example, we observed two distinct neurofibromin isoforms that are differentially expressed in the brain and mammary gland. Both in-frame and premature stop sequence variants resulted in significantly reduced neurofibromin mammary expression, whereas expression of the 250 kD isoform was largely preserved in the brain regardless of *Nf1* status. Collectively, these findings suggest that RAS regulation in the breast is distinct from other tissues, and that breast cancer predisposition may be linked to a unique isoform of neurofibromin that is abundantly expressed in mammary tissue.

One issue that vexed us initially was the divergence of survival between two genotype-matched *Nf1*^*IF-57*^ lines. Analysis of the mRNA in em2 *Nf1*^*IF-57*^ and em3 *Nf1*^*IF-57*^ lines identified an additional deletion in exon 21 in em2 *Nf1*^*IF-57*^. Differential mRNA isoform expression has been observed in breast cancer and may occur through alternate promoter usage, alternate splicing, and alternate 3′UTR usage.^56^ Importantly, differential mRNA isoform expression has been identified and associated with distinct breast cancer subtypes.^56^ Specific to the *NF1* gene, splice variation has also been described.^57,58^ Our data strongly extend these findings as we show that both mutation type (IF and PS) and splice variation lead to more aggressive disease. It is possible that *NF1* isoform expression and splice variation instruct tumor development, and may account for variability in genotype-phenotype correlation.

In summary, we have developed a novel *Nf1* rat model that is invaluable for interrogating deregulated RAS signaling and ER networks in sporadic and NF-related breast cancer. Moreover, we identified a frequent mechanism for RAS deregulation through *NF1* shallow deletion that is present in 25% of sporadic breast cancers. The correlation of *NF1* shallow deletions with poor prognosis and the ER-FOXA1-AR networks indicates that *NF1* may be an important prognostic indicator and therapeutic target in endocrine-resistant breast cancers.

## Methods

### CRISPR/sgRNA design and synthesis, and Cas9 mRNA synthesis

CRISPR targets were identified in exon 20 of the rat *Nf1* gene (NCBI rn5 rat genome) using an online design tool (crispr.mit.edu). A region near the middle of exon 20 was targeted by one guide sequence (5′ CRISPR), GCCCTGTCAGTGAACGCAAA (*GGG*), while a downstream region was targeted by a second guide sequence (3′ CRISPR), CGGTCCATAAATCTGCTGAC (*AGG*). The rn5 rat genome reference sequence and annotation was used for CRISPR target design.

### Rat breeding, microinjection into zygotes and blastocyst culture

Sprague-Dawley rats were purchased from Charles River Laboratories. Female rats were superovulated with injection of 20 IU PMSG, followed by injection of 50 IU HCG and immediate mating to CD Sprague–Dawley studs 48 h later. The pronuclei of fertilized rat zygotes were injected with 40 ng/µL Cas9 mRNA and 20 ng/µL each of two sgRNAs. Surviving eggs were surgically transferred to CD Sprague-Dawley pseudopregnant females. All animal experiments were approved and performed in accordance with institutional IACUC protocols.

### Detecting the presence of indels by PCR-HMA and amplicon sequencing

Genomic DNA from rat-tail biopsies was obtained by phenol extraction. DNA samples were directly used as a PCR template to amplify a region flanking the CRISPR target site. PCRs were set up using the following oligonucleotide primers: Ex20-F 5′-TCAACATGACTGGCTTCCTC-3′ and Ex20-R 5′-CATTGGATACAGAGCAGGACTC–3′ to obtain a 284 bp fragment. The amplicons were subjected to denaturation-slow renaturation to facilitate formation of heteroduplexes using a thermocycler. These samples were then resolved on polyacrylamide gels (10%) and the resulting mobility profiles were used to infer efficiency of CRISPR–Cas9 nuclease activity. PCR using the same primers flanking CRISPR target site (Ex20-F and Ex20-R) was performed to amplify regions with indels (284 bp). The amplified products were cloned using the TOPO-TA cloning kit (Invitrogen, Carlsbad, CA). Ten representative colonies were picked from each plate and grown in 4 mL liquid cultures to isolate plasmid DNA. Plasmid DNA was sequenced using M13F and M13R primers.

### Genotyping

Rat DNA was collected from tail clips by phenol extraction (Invitrogen 15593-031). Target regions of the DNA were PCR amplified using the forward primer rNF1-1A 5′– CTTAGGCTGCAGAAAGTCTTC-3′ and reverse primer rNF1-1B 5′-CTTCACCTGTCCTTGAGAGTC-3′. PCR products were digested with Hpy8I (Thermo Fisher Scientific). After digestion, DNA fragments were separated by electrophoresis on a 2% agarose gel and stained with ethidium bromide. Premature stop (439 bp) and in-frame (185 bp) mutations were identified by gel imaging.

### RT-PCR

RNA was isolated from rat tissue by Trizol extraction (Invitrogen). RNA was treated with DNase I (Thermo Fisher Scientific #18068015). Complementary DNA (cDNA) was prepared by using random primers (Life Technologies 48190-011) and SuperScript™ II Reverse Transcriptase (Invitrogen 18064-022). PCR was performed with forward primer (5′-TCAGTACACAACTTCTTGCC-3′) and reverse primer (5′-GAACACGAACATATCTGACC-3′) to create a 826 bp amplicon. These PCR products were digested with the restriction enzyme Hpy8I as described above to identify mutations. Digested PS amplicons make a 745 bp product and digested wild type make a 390 bp and 368 bp product. Digested PCR products were run on a 10% TGX-PAGE gel (BioRad) and stained with ethidium bromide.

### Histology

Tumors were fixed and processed following the previous methods^59^. DAKO PT Link was used for antigen retrieval. Immunohistochemical staining was done with ER (Thermo MA5-13304), HER2 (Thermo MA5-13105), Cytokeratin (DAKO AE1/AE3), PR (Thermo MA1-411), and pERalpha S118 (SAB4504399 Sigma-Aldrich) using the DAKO Autostainer Link 48. Ki67 (Spring Bioscience SP6) was stained using the Ventana Discovery Ultra.

### Rat Embryo Fibroblast (REF) Isolation

At embryonic day 13.5, embryos were harvested and isolated independently in petri dishes with PBS. The placenta was removed followed by the yolk sac. Embryos were then dissected by removing the head, organs, and blood vessels. The remaining tissue was transferred to a new petri dish and finely chopped using surgical blades. Tissue was then digested in 0.25% trypsin for 15 min at 37 °C. Cells were passed through a cell strainer to isolate single cells. Cells were plated and grown in DMEM (Gibco 11995-065) supplemented with 10% FBS and 1% penicillin.

### Rat mammary epithelial cell (MEC) isolation

Mammary glands from adult rats were dissected and minced. Single-cell suspensions were made by digestion of minced tissue with EpiCultB medium (StemCell Technologies), 5% fetal bovine serum, containing 1:9 collagenase/hyaluronidase cocktail solution (StemCell Technologies) for 15 h at 37 °C. Samples were centrifuged and ammonium chloride (StemCell Technologies) in HBSS + 2% FBS (HF) was added to the pellet to lyse red blood cells. Organoids were isolated by differential centrifugation and were dissociated in 0.2% trypsin-EDTA for 5 min followed by 5 U/ml dispase (StemCell Technologies) with 0.1 mg/ml DNase I (StemCell Technologies) for 5 min. Cells were filtered through a 40 μm cell strainer. To separate epithelial cells from hematopoietic and endothelial cells, samples were subjected to Miltenyi Biotec’s Dead Cell Removal kit followed by immunomagnetic cell selection using a Miltenyi quadroMACS magnet (Miltenyi Biotec). Antibodies for selection included CD31-biotin and CD45-biotin (Miltenyi Biotec). The selection was carried out according to the manufacturer’s protocol using an LS column and anti-biotin microbeads (Miltenyi Biotec) and selection was verified by flow cytometry analysis by comparing the pre (CD45+, CD31+, CD24+, CD29+) and post-selected (CD24+, CD29+) cell populations. To deplete stromal contaminants, eluted cells were plated at 2 × 10^6^ cells/plate on 100 mm plates for three days in DMEM/F12 (Gibco) containing: 10% horse serum (Thermo Fisher Scientific), 20 ng/mL EGF (Sigma), 0.5 μg/mL hydrocortisone (Sigma), 100 ng/mL cholera toxin (Sigma), and 10 ug/mL insulin (Sigma). Minimal fibroblast contamination was verified by microscopically observing the cultures before harvesting.

### Immunoblot analysis

Rat tissue was homogenized with a hand held pellet pestle and rat mammary epithelial cells were lysed in RIPA buffer (20 mM TrisHCL, pH 7.6; 5 mM EDTA; 150 mM NaCl; 0.5% NP-40; 50 mM NaF; 1 mM beta-glycerophosphate) supplemented with PhosSTOP (Roche) and protease inhibitors (Roche). Samples were resolved by SDS-polyacrylamide gel electrophoresis on a 7.5% TGX-PAGE gel (BioRad) and transferred overnight at 4 °C. Blots presented in figure panels were derived from the same experiment and were processed in parallel. Immunoblotting was performed using the following antibodies: neurofibromin H-12 (SC-376886) from Santa Cruz Technology; pERalpha (SAB4504399) from Sigma-Aldrich; ERalpha (MA5-13304) from Thermo; β-actin (#3700), HSP90 (#4877), pERK (#4370), ERK (#9102), pAKT (#9271), AKT (9272), pSTAT3 (#9145), STAT3 (#9139), pMET (#3077) and MET (#3127) from Cell Signaling.

### Immunofluorescence

For immunofluorescence, FFPE tumors and mammary fat pad sections were deparrafinized and underwent heat-induced epitope retrieval (EDTA/borate/Tris) and stained using the Ventana Discovery Ultra (Ventana Medical Systems). Primary antibodies included pERK (#4370), pS6 (#2215), and pSTAT3 (#9145) from Cell Signaling, along with pMET (ab5662) from Abcam. In addition to DAPI, primary antibodies were revealed with anti-rabbit Alexa-Fluor 594 (#8889, Cell Signaling). Lack of non-specific staining of the secondary antibody was confirmed in a secondary-only control. Using a Nikon A1plus-RSi laser scanning confocal microscope, images were taken with a 10× objective. Confocal z-stacks were acquired with a 60× oil immersion objective. PMT levels were set using the control mammary fat pad. Three fields of view per sample were captured with Nikon software. Using FIJI, maximum projection intensity images were generated.

### Statistical analysis

For analysis of *Nf1* rat survival, data were plotted using Kaplan–Meier curves and p-values were calculated using a Cox mixed-effects model with a frailty term for litter (https://CRAN.R-project.org/package=coxme). Linear contrasts with Benjamini–Hochberg false discovery rate adjustments were used to test specific hypotheses. All analyses described below were performed using R v 3.4.0. (https://cran.r-project.org/) All hypotheses were two-sided with a significance level of 0.05. Ki67 analysis was analyzed using a linear mixed-effects regression and a Wald test. Poisson regression, via the base R function glm, was used to determine if premature stop and in-frame deletion rats had significantly different tumor counts at euthanasia. Ovariectomy analysis was analyzed using a multi-level mixed-effects beta regression with a random intercept for tumor, clustered within rat lines. This model was fit using the R package glmmTMB^60^ and the squeeze algorithm.^61^

### METABRIC analysis

Survival analysis was conducted in R v(3.4.2) using the packages survival (https://cran.r-project.org/web/packages/survival/index.html), survminer (https://cran.r-project.org/web/packages/survminer/index.html), and coxme. Multiple variables were assessed (Supplementary Table 4). Clinical, copy number, and gene expression data from the METABRIC data set^33^ were accessed using the cgdsr (https://cran.r-project.org/web/packages/cgdsr/index.html) package in R. Patients were selected based on the availability of both copy number and gene expression data, and HER2 negative status. Geneco-expression network analysis was conducted using the WGCNA (v1.61) package in R to identify gene co-expression modules from mRNA expression data (Illumina Human v3 Microarray).^43^ Data were normalized as previously described. Unsigned correlations were used with a soft threshold value β of 10 and a treecut value of 0.15, a minimum number of genes in the module set to 30. The β and treecut parameters were chosen after assessing the quality of modules detected, and all other parameters used default settings. Unsupervised clustering of WGCNA results was run using the pheatmap package in R (v1.0.8). Logistic regression, via the base R function glm, was used to determine if identified clusters had significantly different rates of shallow deletion mutations. Specific gene networks were visualized using MetaCore from Thomson Reuters (v6.33 build 69110). The RAS and KRAS gene lists used in the analysis were provided by the Broad Institute MSigDB database (v6.1) Hallmark gene set collection and BioCarta (c) (2000–2017 BioCarta, all rights reserved). Module gene lists and processed METABRIC data are available at Synapse (ID: syn11946759).

## Electronic supplementary material

Supplemental Material

## Data Availability

The authors declare that the main data supporting the findings of this study are available within the article and its Supplementary Information files. Extra data are available from the corresponding author upon request.

## Electronic supplementary material

**Supplementary information** accompanies the paper on the *npj Breast Cancer* website (10.1038/s41523-018-0080-8).
