# Supplementary material for: NF1 deficiency correlates with estrogen receptor signaling and diminished survival in breast cancer
Source: NPJ Breast Cancer. 2018 Aug 30;4:29. doi: 10.1038/s41523-018-0080-8 (PMC6117327; doi:10.1038/s41523-018-0080-8)
Supplement: Supplementary file 1 — Supplemental Material [file 41523_2018_80_MOESM1_ESM.pdf]

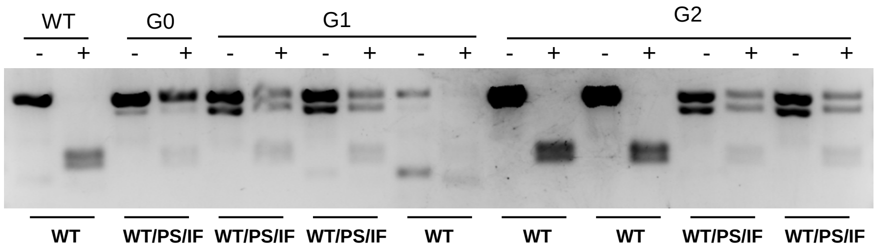

Supplementary Figure 1: Three *Nf1* alleles identified in $Nf1^{IF-63/PS-11}$ line. HMA and sequencing identified 3 *NF1* alleles in $Nf1^{IF-63/PS-11}$: a WT allele, an allele with an 11 bp deletion (premature stop), and an allele with a -63 bp deletion. The presence of more than two alleles was confirmed using unique primer sets in two separate labs. The three alleles were transmitted through the $Nf1^{IF-63/PS-11}$ germline in F1 and F2 generations and did not segregate in either generation as shown in the HMA profiles of individual F1 and F2 $Nf1^{IF-63/PS-11}$ rats.

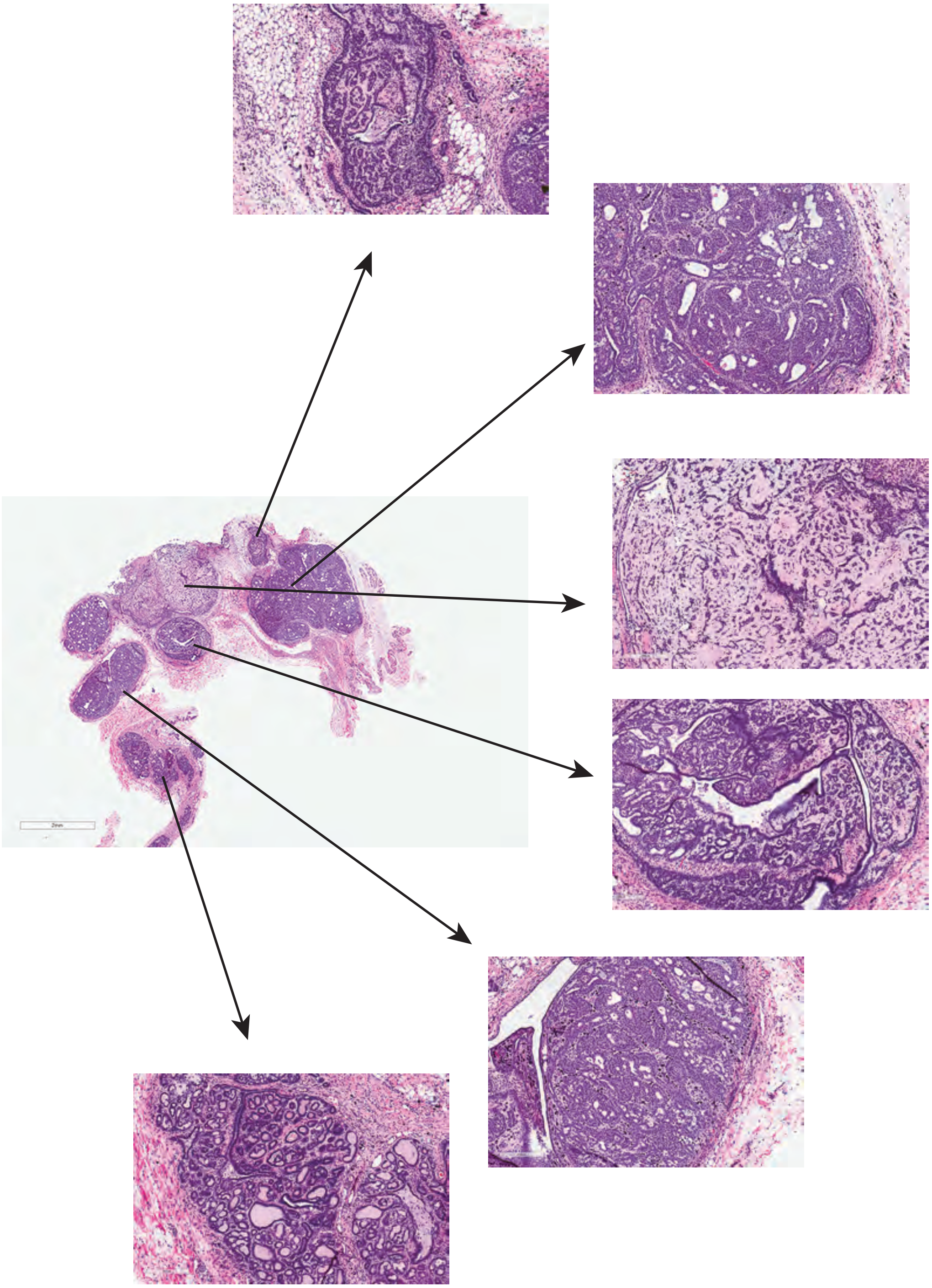

Supplementary Figure 2: Multiple tumors are commonly observed in *Nf1* mammary glands.
The level of tumor burden in the mammary pads of several animals was substantial. Shown above is the 4th mammary pad from *rNf1* #413.

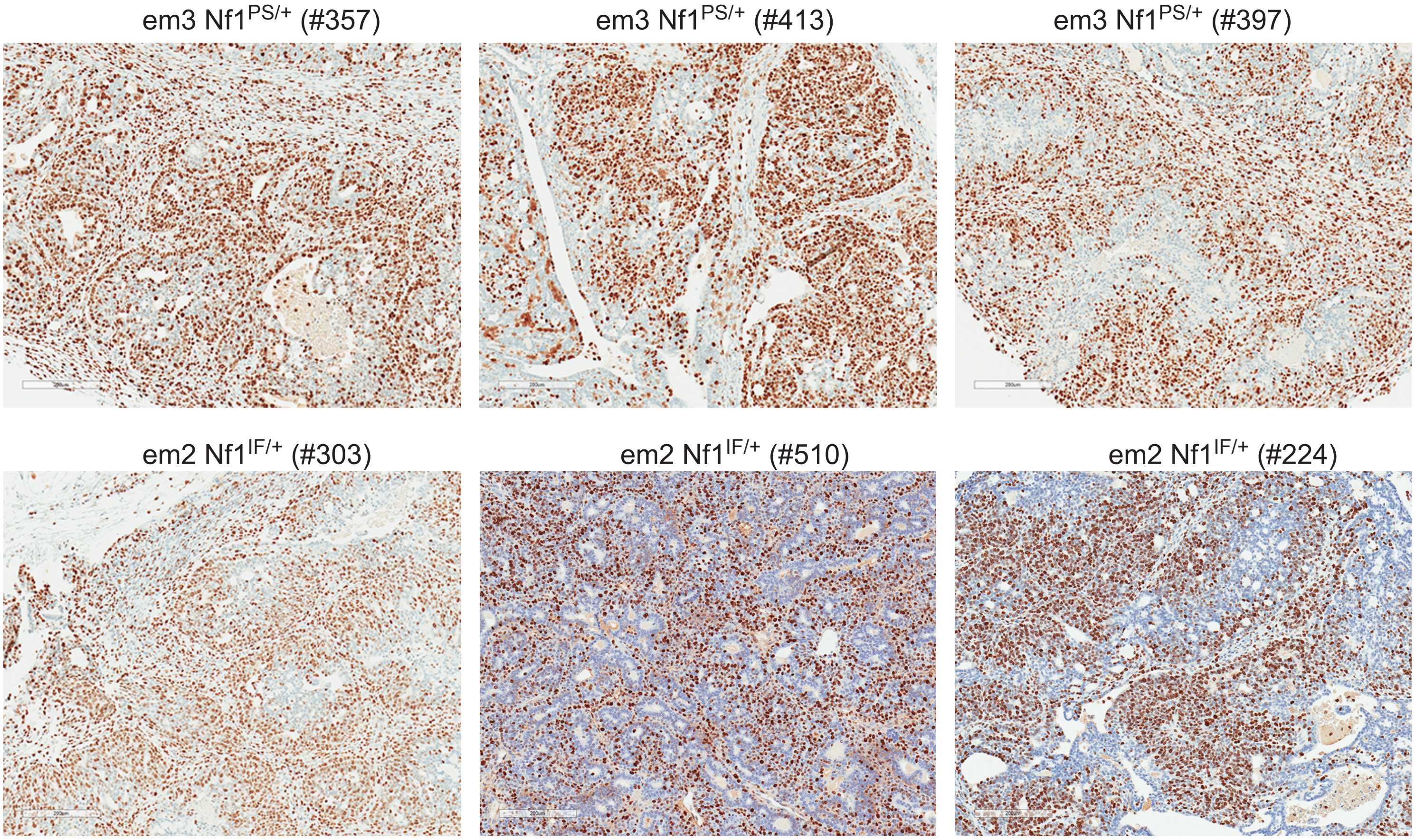

Supplementary Figure 3: Proliferation in *Nf1*$^{IF/+}$ and *Nf1*$^{PS/+}$ tumors. We evaluated Ki67 staining in tumors using the Leica Aperio Nuclear Algorithm and determined there was no significant difference in proliferative staining between *Nf1*$^{IF/+}$ and *Nf1*$^{PS/+}$ tumors. As shown above, Ki67 staining (DAB) was variable among each genotype. Images are shown at 100X magnification.

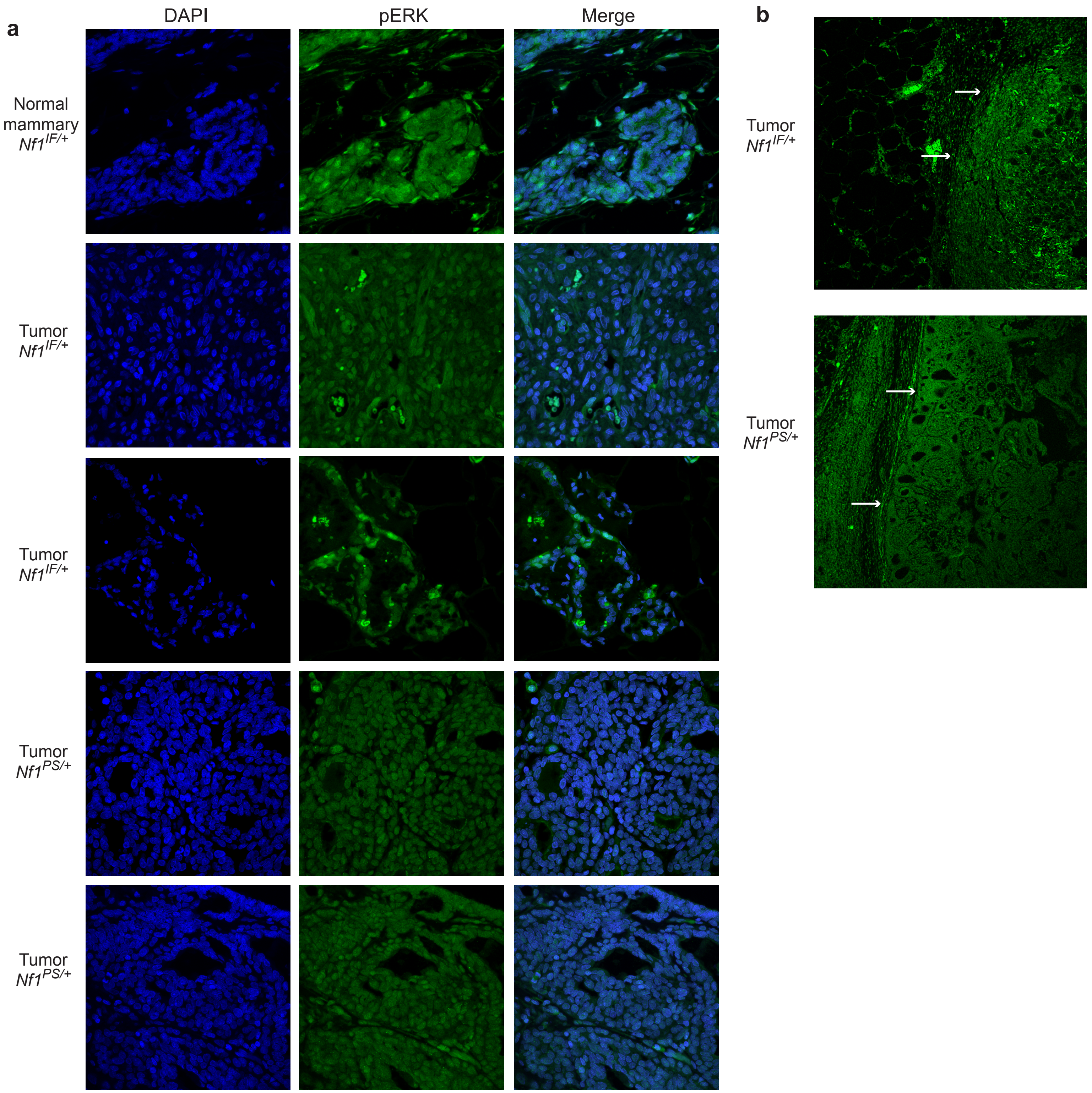

Supplementary Figure 4: Increased nuclear pERK signal is present in *Nf1*-deficient mammary tumors. a) Immunofluorescence of pERK (Thr202/Tyr204) was evaluated in a normal mammary gland and mammary adenocarcinomas from $Nf1^{IF/+}$ and $Nf1^{PS/+}$ rats. In normal tissue, pERK signal was present predominantly in the cytoplasm comared to the nuclear pERK signal present in the tumors. Confocal images are shown at 600X. DAPI was used as a nuclear stain. b) Imaging of tumors at 100X revealed that ERK activatoin is stronger at the tumor periphery (arrows).

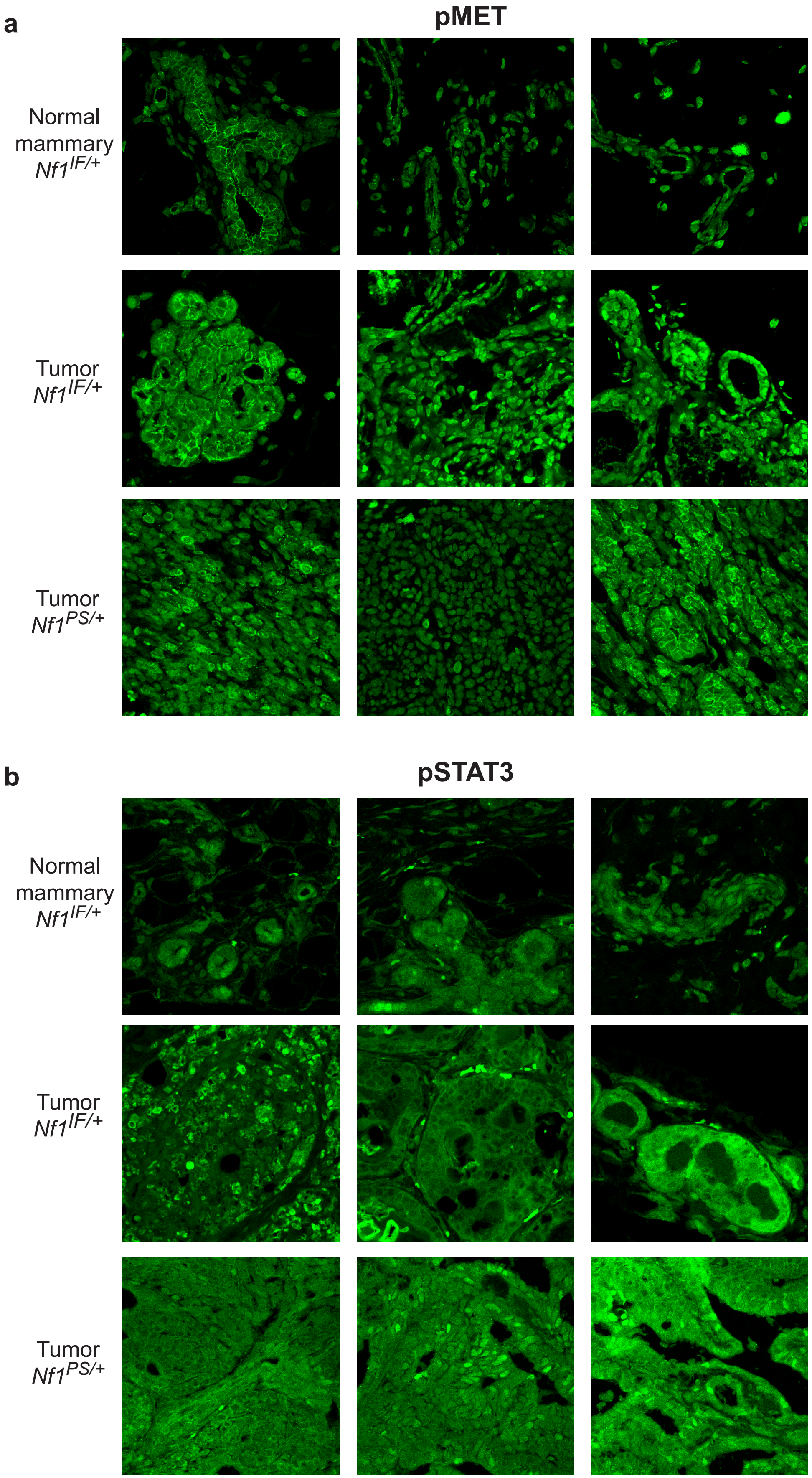

Supplementary Figure 5: Increased pMET and pSTAT3 expression in *Nf1*-deficient mammary tumors. Immunofluorescence of a) pMET (Y1234/35) and b) pSTAT3 (Y705) was evaluated in a normal mammary gland and mammary adenocarcinomas from $Nf1^{IF/+}$ and $Nf1^{PS/+}$ rats. Confocal images are shown at 600X.

<table>
<thead>
<tr><th>#</th><th>Sex</th><th>5' CRISPR</th><th>3' CRISPR</th><th>Tumor Onset (days)</th><th>Age of Death (days)</th><th>F1</th><th>DNA Indels</th><th>Line Name</th></tr>
</thead>
<tbody>
<tr><td>1</td><td>M</td><td>wt</td><td>wt</td><td></td><td>Alive</td><td>NA</td><td></td><td></td></tr>
<tr><td>2</td><td>M</td><td colspan="2">-57 (wt)</td><td>NA</td><td>477</td><td>Yes</td><td>c.2669_2725del</td><td>$\mathbf{\mathit{Nf1}}^{IF-57/+}$</td></tr>
<tr><td rowspan="2">3</td><td rowspan="2">M</td><td>+1,-8 bp</td><td>+4,-10</td><td rowspan="2">NA</td><td rowspan="2">416</td><td rowspan="2">Yes</td><td rowspan="2">c.2659_2660insT,2660_2667del,2715_2716insTGTT,2716-2725del
c.2669_2725del</td><td rowspan="2">$\mathbf{\mathit{Nf1}}^{IF-57/PS-8}$</td></tr>
<tr><td colspan="2">-57</td></tr>
<tr><td>4</td><td>F</td><td colspan="2">-61 (wt)</td><td>53</td><td>58</td><td>NA</td><td></td><td></td></tr>
<tr><td>5</td><td>F</td><td colspan="2">IF indel (HMA)</td><td>53</td><td>63</td><td>NA</td><td></td><td></td></tr>
<tr><td>6</td><td>F</td><td colspan="2">-54</td><td>60</td><td>77</td><td>*</td><td></td><td></td></tr>
<tr><td rowspan="2">7</td><td rowspan="2">M</td><td>+1,-4</td><td>-20</td><td rowspan="2"></td><td rowspan="2">Alive</td><td rowspan="2">None</td><td rowspan="2"></td><td rowspan="2"></td></tr>
<tr><td colspan="2">-66</td></tr>
<tr><td>8</td><td>F</td><td>-11 (wt)</td><td>-4</td><td></td><td>62$^{\#}$</td><td>NA</td><td></td><td></td></tr>
<tr><td>9</td><td>F</td><td>+8,-1</td><td>-11</td><td>29</td><td>49</td><td>NA</td><td></td><td></td></tr>
<tr><td rowspan="2">10</td><td rowspan="2">F</td><td>-11</td><td></td><td rowspan="2">51</td><td rowspan="2">64</td><td rowspan="2">Yes</td><td rowspan="2">c.2661_2671del
c.2662_2724del</td><td rowspan="2">$\mathbf{\mathit{Nf1}}^{IF-63/PS-11}$</td></tr>
<tr><td colspan="2">-63 (wt)</td></tr>
<tr><td>11</td><td>M</td><td>-57</td><td></td><td></td><td>314</td><td>None</td><td></td><td></td></tr>
<tr><td rowspan="2">12</td><td rowspan="2">F</td><td>-6</td><td>-10</td><td rowspan="2">47</td><td rowspan="2">62</td><td rowspan="2">*</td><td rowspan="2"></td><td rowspan="2"></td></tr>
<tr><td colspan="2">-54</td></tr>
<tr><td rowspan="2">13</td><td rowspan="2">F</td><td>-11</td><td>-4</td><td rowspan="2">54</td><td rowspan="2">198</td><td rowspan="2">Yes</td><td rowspan="2">c.2661_2671del,2721_2724del
c.2669_2722del</td><td rowspan="2">$\mathbf{\mathit{Nf1}}^{IF-54/PS-11}$</td></tr>
<tr><td colspan="2">-54</td></tr>
<tr><td rowspan="2">14</td><td rowspan="2">F</td><td>-17</td><td></td><td rowspan="2">47</td><td rowspan="2">68</td><td rowspan="2">NA</td><td rowspan="2"></td><td rowspan="2"></td></tr>
<tr><td colspan="2">-54</td></tr>
<tr><td>15</td><td>M</td><td colspan="2">-66 (wt)</td><td></td><td>Alive</td><td>NA</td><td></td><td></td></tr>
<tr><td>16</td><td>M</td><td colspan="2">IF indel (HMA)</td><td></td><td>Alive</td><td>NA</td><td></td><td></td></tr>
<tr><td rowspan="2">17</td><td rowspan="2">M</td><td>-2 (wt)</td><td></td><td rowspan="2"></td><td rowspan="2">Alive</td><td rowspan="2">NA</td><td rowspan="2"></td><td rowspan="2"></td></tr>
<tr><td colspan="2">-66</td></tr>
<tr><td rowspan="2">18</td><td rowspan="2">F</td><td colspan="2">-66</td><td rowspan="2">28</td><td rowspan="2">54</td><td rowspan="2">NA</td><td rowspan="2"></td><td rowspan="2"></td></tr>
<tr><td>-11</td><td>3sub,2sub</td></tr>
<tr><td>19</td><td>F</td><td></td><td>+1 (wt)</td><td>89</td><td>189</td><td>NA</td><td></td><td></td></tr>
</tbody>
</table>

Supplementary Table 1: Summary of G0 animals obtained from CRISPR-Cas9 injections. Summary of indels resulting from CRISPR-Cas9 injections including tumor onset and survival. The most common base pair alterations at the 5' and 3' CRISPR sites are listed (additional indels that were identified in each G0 animal are listed in Supplemental Table 2). Mutant alleles that resulted in large deletions between 5' and 3' CRISPR target regions are listed in the middle of the 5' and 3' CRISPR columns; "wt" refers to the wildtype allele being observed by sequencing. IF indel (HMA) indicates that indels were identified by HMA but not sequence verified. All females were euthanized due to tumor burden except for #8 that was taken for control tissue (notated with #). DNA indel notations for non-founder animals can be found in Supplementary Table 2. * Euthanized when pregnant due to tumor burden. None = bred but no progeny.

ID	Sequence (5′-3′) 5′ CRISPR > 3′ CRISPR <	Indels (Clones)	Protein	DNA
**WT**	TACAGCCCACCTATGGGCCCTGTCAGTGAACGCAAAGGGTCTATGATTTCTGTAATGTCTTCTGAAGGGAATGTTGATTCACCTGTCAGCAGATTTATGGACCGGCTTCTGTCCTTGATGGTG			
	Y S P P M G P V S E R K G S M I S V M S S E G N V D S P V S R F M D R L L S L M V			
**1**	TACAGCCCACCTATGGGCCCTGTCAGTGAACGCAAAGGGTCTATGATTTCTGTAATGTCTTCTGAAGGGAATGTTGATTCACCTGTCAGCAGATTTATGGACCGGCTTCTGTCCTTGATGGTG	WT (4)		
	Y S P P M G P V S E R K G S M I S V M S S E G N V D S P V S R F M D R L L S L M V			
**2**	TACAGCCCACCTATGGGCCCTGTCAGTGAACGCA---GATTTATGGACCGGCTTCTGTCCTTGATGGTG	WT(4);-57(4)	In frame deletion	c.2669_2725del
	Y S P P M G P V S E R R F M D R L L S L M V C	19 aa deletion		
**3**	TACAGCCCACCTATGGGCCCTGTCAT-------AAAGGGTCTATGATTTCTGTAATGTCTTCTGAAGGGAATGTTGATTCATGTT----------GATTTATGGACCGGCTTCTGTCCTTGATG<br>GTG	WT(0);+1,-8,+4,-10(3);	Premature stop	c.2659_2660insT,2660_2667del,2715_2716insTGTT,2716-2725del
	Y S P P M G P V I K G L *	4 aa* + stop		
	TACAGCCCACCTATGGGCCCTGTCAGTGAACGCA---GATTTATGGACCGGCTTCTGTCCTTGATGGTG	-57(1)	In frame deletion	c.2669_2725del
	Y S P P M G P V S E R R F M D R L L S L M V C	19 aa deletion		
**4**	TACAGCCCACCTATGGGCCCTGTCAGTGAA---GATTTATGGACCGGCTTCTGTCCTTGATGGTG	WT(2);-61(4)	Premature stop	c.2665_2725del
	Y S P P M G P V S E D L W T G F C P *	8 aa* + stop		
**6**	TACAGCCCACCTATGGGCCCTGTCAGTGAACGCA--GCAGATTTATGGACCGGCTTCTGTCCTTGATGGTG	WT(0);-54(2)	In frame deletion	c.2669_2722del
	Y S P P M G P V S E R S R F M D R L L S L M V	18 aa deletion		
**7**	TACAGCCCACCTATGGGCCCTGTCAGTGAACT----AGGGTCTATGATTTCTGTAATGTCTTCTGAAGGGAATGTTGATTCACC--------------------GGCTTCTGTCCTTGATGGTG	WT(0);+1,-4,-20(4)	Premature stop	c.2665_2666insT,2666_2669del
	Y S P P M G P V S E L G S M I S V M S S E G N V D S P A S V L D G V *	23 aa* + stop		
	TACAGCCCACCTATGGGCCCTGTCAG--ATTTATGGACCGGCTTCTGTCCTTGATGGTG	-66(3)	deletion	c.2661_2726del
	Y S P P M G P V R F M D R L L S L M V C N H E	22 aa deletion		
	TACAGCCCACCTATGGACCCTGTCAG--ATTTATGGACCGGCTTCTGTCCTTGATGGTG	1sub,-66(1)	Missense/In frame deletion	c.2651G>A,2661_2726del
	Y S P P M D P V R F M D R L L S L M V C N H E	1 aa* + 22 aa deletion		
	TACAGCCCACCTATGGGCCCTGTCAG-----------GGTCTATGATTTCTGTAATGTCTTCTGAAGGGAATGTTGATTCACC--------------------GGCTTCTGTCCTTGATGGTG	-11,-20(1)	Premature stop	c.2661_2671del,2718_2737del
	Y S P P M G P V R V Y D F C N V F *	9 aa* + stop		
**8**	TACAGCCCACCTATGGGCCCTGTCAG-----------GGTCTATGATTTCTGTAATGTCTTCTGAAGGGAATGTTGATTCACCTG----CAGATTTATGGACCGGCTTCTGTTCTTGATGGTG	WT(3) & -11,-4,1sub (1)	Premature stop	c.2661_2671del,2720_2723del,2747C>T
	Y S P P M G P V R V Y D F C N V F *	9 aa* + stop		
	TACAGCCCACCTATGGGCCCTGTCAGTGAACGCAAAGGGTTTATGATTTCTGTAATGTCTTCTGAAGGGAATGTTGATTCACCTGTCAGCAGATTTATGGACCGGCTTCTGTCCTTGATGGTG	1sub(1)	Missense	c.2675C>T
	Y S P P M G P V S E R K G F M I S V M S S E G	1 aa*		
	TACAGCCCACCTATGGGCCCTGTCAG------------GTCTATGATTTCTGTAATGTCTTCTGAAGGGAATGTTGATTCACCTGTCAGCAGATTTATGGACCGGCTTCTGTCCTTGATGGTG	-12(1)	deletion	c.2661_2672del
	Y S P P M G P V R S M I S V M S S E G N V D S	4 aa deletion		
	TACAGCCCACCTATGGGCCCTGTCAG-----------GGTCTATGATTTCTGTAATGTCTTCTGAAGGGAATGTTGATTCACCTG----CAGATTTATGGACCGGCTTCTGTCCTTGATGGTG	-11,-4(1)	Premature stop	c.2661_2671del,2720_2723del
	Y S P P M G P V R V Y D F C N V F *	9 aa* + stop		
	TACAGCCCACCTATGGGCCCTATCAG-----------GGTCTATGATTTCTGTAATGTCTTCTGAAGGGAATGTTGATTCACCTG----CAGATTTATGGACCGGCTTCTGTCCTTGATGGTG	1sub,-11,-4(1)	Premature stop	c.2656G>A,2661_2671del2720_2723del
	Y S P P M G P I R V Y D F C N V F *	10 aa* + stop		
**9**	TACAGCCCACCTATGGGCCCTGTCAGTGAACGGTCTATGT-AAAGGGTCTATGATTTCTGTAATGTCTTCTGAAGGGAATGTTGATTCACCT-----------TATGGACCGGCTTCTGTCCT<br>TGATGGTG	WT(0);+8,-1,-11(2)	Premature stop	c.2666_2667insGTCTATGT,2667del,2719_2729del
	Y S P P M G P V S E R S M *	2 aa* + stop		
**10**	TACAGCCCACCTATGGGCCCTGTCAG-----------GGTCTATGATTTCTGTAATGTCTTCTGAAGGGAATGTTGATTCACCTGTCAGCAGATTTATGGACCGGCTTCTGTCCTTGATGGTG	WT(1);-11(1)	Premature stop	c.2661_2671del
	Y S P P M G P V R V Y D F C N V F *	9 aa* + stop		
	TACAGCCCACCTATGGGCCCTGTCAGT---AGATTTATGGACCGGCTTCTGTCCTTGATGGTG	-63(2)	deletion	c.2662_2724del

	Y S P P M G P V S R F M D R L L S L M V C N H	21 aa deletion		
**11**	TACAGCCCACCTATGGGCCCTGTCAGTGAACGCA---GATTTATGGACCGGCTTCTGTCCTTGATGGTG	WT(0);-57(4)	deletion	c.2669_2725del
	Y S P P M G P V S E R R F M D R L L S L M V C	19 aa deletion		
**12**	TACAGCCCACCTATGGGCCCTGTCAGTGAACGCA--GCAGATTTATGGACCGGCTTCTGTCCTTGATGGTG	WT(0);-54 (1)	deletion	c.2669_2722del
	Y S P P M G P V S E R S R F M D R L L S L M V	18 aa deletion		
	TACAGCCCACCTATGGGCCCTGTCAGTGAA------GGGTCTATGATTTCTGTAATGTCTTCTGAAGGGAATGTTGATTCACCTGTCAGCAGATTTATGGACCGGCTTCTGTCCTTGATGGTG	-6(1)	deletion	c.2665_2670del
	Y S P P M G P V S E G S M I S V M S S E G N V	2 aa deletion		
	TACAGCCCACCTATGGGCCCTGTCAGTGAACGC**C**AAAGGGTCTATGATTTCTGTAATGTCTTCTGAAGGGAATGTTGATTCA----------GATTTATGGACCGGCTTCTGTCCTTGATGGTG	+1,-10(2)	Premature stop	c.2667_2688insC,2726_2725del
	Y S P P M G P V S E R Q R V Y D F C N V F *	10 aa* + stop		
**13**	TACAGCCCACCTATGGGCCCTGTCAGTGAACGCA--GCAGATTTATGGACCGGCTTCTGTCCTTGATGGTG	WT(0);-54(1)	deletion	c.2669_2722del
	Y S P P M G P V S E R S R F M D R L L S L M V	18 aa deletion		
	TACAGCCCACCTATGGGCCCTGTCAG-----------GGTCTATGATTTCTGTAATGTCTTCTGAAGGGAATGTTGATTCACCTGT----AGATTTATGGACCGGCTTCTGTCCTTGATGGTG	-11,-4(4)	Premature stop	c.2661_2671del,2721_2724del
	Y S P P M G P V R V Y D F C N V F *	9 aa* + stop		
**14**	TACAGCCCACCTATGG-CCCTGTCAGTGA-----------------TTTCTGTAATGTCTTCTGAAGGGAATGTTGATTCACCTGTCAGCAGATTTATGGACCGGCTTCTGTCCTTGATGGTG	WT(0);-1,-17(1)	Premature stop	c.2651del,2664_2680del
	Y S P P M A L S V I S V M S S E G N D S P V S R F M D R L L S L M V C N H E K V G	> 33 aa* + stop?		
	TACAGCCCACCTATGGGCCCTGTCAGTGA-----------------TTTCTGTAATGTCTTCTGAAGGGAATGTTGATTCACCTGTCAGCAGATTTATGGACCGGCTTCTGTCCTTGATGGTG	-17(1)	Premature stop	c.2664_2680del
	Y S P P M G P V S D F C N V F *	7 aa* + stop		
	TACAGCCCACCTATGGGCCCTGTCAGTGAACGCA--GCAGATTTATGGACCGGCTTCTGTCCTTGATGGTG	-54(1)	deletion	c.2669_2722del
	Y S P P M G P V S E R S R F M D R L L S L M V	18 aa deletion		
**15**	TACAGCCCACCTATGGGCCCTGTCAG--ATTTATGGACCGGCTTCTGTCCTTGATGGTG	WT(3);-66(2)	deletion	c.2661_2726del
	Y S P P M G P V R F M D R L L S L M V C N H E	22 aa deletion		
**16**	TACAGCCCACCTATGGGCCCTGTCAGTGAACGCAAAGGGTCTATGATTTCTGTAATGTCTTCTGAAGGGAATGTTGATTCACCTGTCAGCAGATTTATGGACCGGCTTCTGTCCTTGATGGTG	WT(3)		
	Y S P P M G P V S E R K G S M I S V M S S E G N V D S P V S R F M D R L L S L M V			
**17**	TACAGCCCACCTATGGGCCCTGTCAGTGAAC--AAAGGGTCTATGATTTCTGTAATGTCTTCTGAAGGGAATGTTGATTCACCTGTCAGCAGATTTATGGACCGGCTTCTGTCCTTGATGGTG	WT(1);-2 (1)	Premature stop	c.2666_2667del
	Y S P P M G P V S E Q R V Y D F C N V F *	10 aa* + stop		
	TACAGCCCACCTATGGGCCCTGTCAG--ATTTATGGACCGGCTTCTGTCCTTGATGGTG	-66 (1)	deletion	c.2661_2726del
	Y S P P M G P V R F M D R L L S L M V C N H E	22 aa deletion		
	TACGGCCCACCTATGGGCCCTGTCAGTGAA---AAAGGGTCTATGATTTCTGTAATGTCTTCTGAAGGGAATGTTGATTCACCTGTCAGCAGATTTATGGACCGGCTTCTGTCCTTGATGGTG	1sub,-3(1)	Premature stop?	c.2638A>G,2665_2667del
	Y G P P M G P V S E K G S M I S V M S S E G N	< 33 aa* + stop?		
	TACAGCCCACCTATGGGCCCTGTCAGTGAA---AAAGGGTCTATGATTTCTGTAATGTCTTCTGAAGGGAATGTTGATTCACCTGTCAGCAGATTTATGGACC**A**GCTTCTGTCCTTGATGGTG	-3,1sub(1)	In frame deletion/missense	c.2665_2667del,2738G>A
	Y S P P M G P V S E K G S M I S V M S S E G N V D S P V S R F M D Q L L S L M V C	3 aa deletion + 1 aa*		
**18**	TACAGCCCACCTATGGGCCCTGTCAG--ATTTATGGACCGGCTTCTGTCCTTGATGGTG	WT(0);-66 (3)	deletion	c.2661_2726del
	Y S P P M G P V R F M D R L L S L M V C N H E	22 aa deletion		
	TACAGCCCACCTATGGGCCCTGTCAG-----------GGTCTATGATTTCTGTAATGTCTTCTGAAGGGAATGTTGATTCACCTG**AAT**G**TT**GATTTATGGACCGGCTTCTGTCCTTGATGGTG	-11,3sub,2sub (1)	Premature stop	c.2661_2671del,2720T>A,2721C>A,2722A>T,2724C>T,2725A>T
	Y S P P M G P V R V Y D F C N V F *	9 aa* + stop		
**19**	TACAGCCCACCTATGGGCCCTGTCAGTGAACGCAAAGGGTCTATGATTTCTGTAATGTCTTCTGAAGGGAATGTTGATTCACCTGTCA**A**GCAGATTTATGGACCGGCTTCTGTCCTTGATGGTG	WT(1);+1(2)	Premature stop	c.2722_2723insA
	Y S P P M G P V S E R K G S M I S V M S S E G N V D S P V K Q I Y G P A S V L D G V *	12 aa* + stop		

Supplementary Table 2: Detailed sequence analysis of *Nf1* G0 rats. 34 mutant alleles, including 25 unique mutant alleles, were identified by Sanger sequencing. Sequences for all alleles identified in G0 animals are shown.

G0/F0 Animals

Animal	Sex	Genotype	Age (days)	Tissue/Mammary Pad Description	Pathology
2	M	$Nf1^{IF-57/+}$	477	R1 Mammary Fat Pad	mammary adenocarcinoma - acinar and mucinous
				L4 Mammary Fat Pad	mammary pad - galactorrhea
				R2 Mammary Fat Pad	mammary adenocarcinoma - acinar and ductular
				L1 Mammary Fat Pad	two mammary adenocarcinomas 1-acinar and ductular 2-secretory acinar
				L4 Mammary Fat Pad	mammary pad - galactorrhea
				R1 Mammary Fat Pad	mammary pad - galactorrhea
3	M	$Nf1^{IF-57/PS-8}$	416	L1 Mammary Fat Pad	mammary adenoma - cystic
				Right flank	fibrous histiocytic sarcoma
				L1 Mammary Fat Pad	mammary adenocarcinoma with two small intraductal tumors
				L4 Mammary Fat Pad	mammary adenoma - cystic
4	F		58	R1 Mammary Fat Pad	mammary adenocarcinoma - acinar
				L4 Mammary Fat Pad	mammary adenocarcinoma - acinar and solid
				R4 Mammary Fat Pad	mammary adenocarcinoma - acinar and solid
				L5/6 Mammary Fat Pad	mammary adenocarcinoma - acinar and solid
				L4 Mammary Fat Pad	mammary adenocarcinoma - acinar and solid
6	F	$Nf1^{IF-54/+}$	77	R2 Mammary Fat Pad	mammary adenocarcinoma - acinar
				R5/6 Mammary Fat Pad	mammary adenocarcinoma - acinar
				L2 Mammary Fat Pad	mammary adenocarcinoma - acinar
				L4 Mammary Fat Pad	mammary hyperplasia, mammary adenocarcinoma - acinar and ductular
				R5/6 Mammary Fat Pad	mammary hyperplasia; mammary adenocarcinoma - acinar, ductular and cystic
				L5/6 Mammary Fat Pad	mild mammay hyperplasia; mammary adenocarcinoma - cystic and papillary
				R4 Mammary Fat Pad	mammary adenocarcinoma - ductular
				L4/5 Mammary Pad	moderately severe acinar hyperplasia
10	F	$Nf1^{IF-63/PS-11/+}$	64	R4 Mammary Fat Pad	mammary adenocarcinoma - acinar and solid
				R5/6 Mammary Fat Pad	lactating mammary gland with small acinar adenocarcinoma
				R5/6 Mammary Fat Pad	lactating mammary with adenocarcinoma - acinar and cystic
				L4 Mammary Fat Pad	mammary adenocarcinoma - acinar and solid
				L5/6 Mammary Fat Pad	mammary adenocarcinoma - acinar and solid
				L5/6 Mammary Fat Pad	mammary adenocarcinoma - acinar
13	F	$Nf1^{IF-54/PS-11}$	198	R4 Mammary Fat Pad	mammary adenocarcinoma - acinar and solid
				L5/6 Mammary Fat Pad	2 mammary adenocarcinomas - acinar and ductular 2) acinar
				L3 Mammary Fat Pad	mammary adenocarcinoma - acinar and ductular
				Right Brachial Lymph Node	large lymph node with hyperplasia
				Left Brachial Lymph Node	large lymph node with hyperplasia
				Aortic Lymph Node	lymph node
				L4 Mammary Fat Pad	lymph node

rNF1-2 F1 animals

Folder	Sex	Genotype	Age (days)	Tissue/Mammary Pad Description	Pathology
303	F	$Nf1^{IF-57/+}$	62	R4 Mammary Fat Pad	mammary adenocarcinoma - acinar and solid
				R3 Mammary Fat Pad	mammary adenocarcinoma - solid, ductular with stroma
				L4 Mammary Fat Pad	slight acinar hyperplasia
				L4 normal mammary tissue	mild acinar hyperplasia
304	F	$Nf1^{IF-57/+}$	89	R4 Mammary Fat Pad	mammary adenocarcinoma - acinar and cystic
				L5/6 Mammary Fat Pad	mammary adenocarcinoma - solid, acinar and cystic
				L2 Mammary Fat Pad	mammary adenocarcinoma - solid and cystic
				L1 Mammary Fat Pad	mammary adenocarcinoma - solid and cystic
366	F	$Nf1^{IF-57/+}$	98	L4 Mammary Fat Pad - 1	mammary adenocarcinoma - acinar and ductular
				L4 Mammary Fat Pad - 2	mammary adenocarcinoma - acinar and ductular
				R2 Mammary Fat Pad	mammary adenocarcinoma - acinar and ductular
				R4 Mammary Fat Pad -1	mammary adenocarcinoma - acinar and ductular
				R4 Mammary Fat Pad - 2	mammary adenocarcinoma - cystic and ductular
				L2 Mammary Fat Pad	mammary adenoma

rNF1-3 F1 animals

Folder	Sex	Genotype	Age (days)	Tissue/Mammary Pad Description	Pathology
357	F	$Nf1^{PS-8/+}$	84	R5/6 Mammary Fat Pad	mammary adenocarcinoma - mostly solid with some invasion
				R1 Mammary Fat Pad	mammary adenocarcinoma - acinar
				L4 Mammary Fat Pad - 2	mammary adenocarcinoma - mostly solid with hemorrhage
				L4 Mammary Fat Pad - 1	mammary adenocarcinoma - solid
				L2 Mammary Fat Pad	mammary adenocarcinoma - acinar and solid
				R4 Mammary Fat Pad	mammary adenocarcinoma - acinar and solid
				R3/R4 Normal Mammary Tissue	small mammary intraductal solid adenocarcinoma
413	F	$Nf1^{PS-8/+}$	105	L2 Mammary Fat Pad	mammary adenocarcinoma - acinar
				L4 Mammary Fat Pad - 1	mammary adenocarcinoma - acinar and ductular
				L3 Mammary Fat Pad	mammary adenocarcinoma - acinar and solid
				L4 Mammary Fat Pad - 2	mammary adenocarcinoma - acinar and solid
				L4 Mammary Fat Pad - 3	6 separate mammary tumors, 4 acinar, one adenoma and one with edema
				L5/6 Mammary Fat Pad	solid, acinar, ductular and cystic
				R2 Mammary Fat Pad	5 small mammary acinar tumors in an abscess.
				R5/6 Mammary Fat Pad	mammary adenocarcinoma - ductular, solid and acinar
				R4 Mammary Fat Pad	mammary adenocarcioma - acinar, ductular, cystic

rNF1-10 F1 animals					
Folder	Sex	Genotype	Age (days)	Tissue/Mammary Pad Description	Pathology
246	F	*Nf1* $^{IF-63/PS-11/+}$	96	R2 Mammary Fat Pad	mammary adenocarcinoma - ductular and solid
				L5/6 Mammary Fat Pad - 1	mammary adenocarcinoma - cystic, papillary, solid, and ductal
				L5/6 Mammary Fat Pad -2	mammary adenocarcinoma - ductular, acinar and cystic
				R4 Normal Mammary Tissue	mild acinar hyperplasia
252	F	*Nf1* $^{IF-63/PS-11/+}$	183	R4 Mammary Fat Pad	normal mammary gland
				L5/6 Mammary Fat Pad	mammary adenocarcinoma - acinar
				L4 Mammary Fat Pad	focal hyperplasia and mammary adenoma
				L2 Mammary Fat Pad	mammary adenocarcinoma - solid
				L2 Mammary Fat Pad	normal mammary

rNF1-13 F1 animals					
Folder	Sex	Genotype	Age (days)	Tissue/Mammary Pad Description	Pathology
262	F	*Nf1* $^{PS-11/+}$	92	R2 Mammary Fat Pad	mammary adenocarcinoma - solid, ductular with stroma
				Normal mammary tissue	normal mammary tissue
391	F	*Nf1* $^{PS-11/+}$	89	R4 Mammary Fat Pad - 1	mammary adenocarcinoma - ductular
				R4 Mammary Fat Pad - 2	mammary adenocarcinoma - ductular and solid
				R4 Mammary Fat Pad - 3	mammary adenocarcinoma - ductular
				L4 Mammary Fat Pad	3 small mammary adenocarinomas - acinar
				L2 Mammary Fat Pad	mammary adenocarcinoma - acinar and solid
				R2 Normal Mammary	normal mammary gland
397	F	*Nf1* $^{PS-11/+}$	118	L2 Mammary Fat Pad	mammary adenocarcinoma - solid, acinar and cystic
				L4 Mammary Fat Pad - 2	mammary adenocarcinoma - acinar and solid
				L5/6 Mammary Fat Pad	mammary adenocarcinoma - solid
				R5/6 Mammary Fat Pad	mammary adenocarcinoma - solid and acinar
				L4 Mammary Fat Pad - 1	mammary adenocarcinoma - ductular
				R4 Normal Mammary	mildly hyperplastic mammary gland

Supplementary Table 3: Histopathology of *rNf1* animals

	Shallow Deletion	Diploid	P-value
Sample Size	379	922	
Mean (SD)			
Age at Diagnosis*	55.8 (13)	58.9 (12.2)	< 0.0001
Tumor Size*	29 (20.6)	25 (14.6)	< 0.0001
Median (Q3-Q1)			
NPI†	4.07 (5.06-4.03)	4.04 (5.04-3.04)	< 0.0001
Percentages			
Grade†			< 0.0001
1	5.4%	10.7%	
2	27.3%	43.4%	
3	67.3%	45.9%	
Stage†			0.002
0	0.4%	1%	
1	28.3%	37.9%	
2	57.2%	53.4%	
3	13.4%	7%	
4	0.7%	0.7%	
Subtype‡			
Basal	23%	8.4%	< 0.0001
claudin-low	11.9%	13%	0.57
Her2	18.2%	8.1%	< 0.0001
LumA	19%	38%	< 0.0001
LumB	20.8%	23.2%	0.35
Normal	6.9%	9%	0.21
Not Classified	0.3%	0.3%	
Inferred Menopause‡			0.0001
Pre-	35.1%	24.6%	
Post-	64.9%	75.4%	
ER‡			< 0.0001
Negative	44.1%	21.4%	
Positive	55.9%	78.6%	
Cellularity†			< 0.0001
low	7.9%	11.7%	
moderate	31%	41.8%	
high	61.1%	46.5%	

Supplementary Table 4. Clinical and demographic characteristics for patients with shallow deletion *NF1* mutations and diploids. *Differences between shallow deletion and diploid mutations were tested using a t-test. †Differences between shallow deletion and diploid mutations were tested using a Wilcox test. ‡ Differences between shallow deletion and diploid mutations were tested using a logistic regression.
